# *SLC27A2* marks lipid peroxidation in nasal epithelial cells driven by type 2 inflammation in chronic rhinosinusitis with nasal polyps

**DOI:** 10.1038/s12276-025-01440-1

**Published:** 2025-04-07

**Authors:** Jaewoo Park, Jung Yeon Jang, Jeong Heon Kim, Se Eun Yi, Yeong Ju Lee, Myeong Sang Yu, Yoo-Sam Chung, Yong Ju Jang, Ji Heui Kim, Kyuho Kang

**Affiliations:** 1 Department of Biological Sciences and Biotechnology, Chungbuk National University, Cheongju, Republic of Korea; 2 Department of Otorhinolaryngology–Head and Neck Surgery, Asan Medical Center, University of Ulsan College of Medicine, Seoul, Republic of Korea

**Keywords:** Predictive markers, Translational immunology

## Abstract

Chronic rhinosinusitis with nasal polyps (CRSwNP) is characterized by persistent inflammation and epithelial cell dysfunction, but the underlying molecular mechanisms remain poorly understood. Here we show that dysregulated lipid metabolism and increased lipid peroxidation in nasal polyp epithelial cells contribute to the pathogenesis of CRSwNP. Integrated analysis of bulk and single-cell RNA sequencing data reveals upregulation of *SLC27A2*/FATP2 in nasal polyp epithelium, which correlates with increased lipid peroxidation. *SLC27A2*-positive epithelial cells exhibit enriched expression of lipid peroxidation pathway genes and enhanced responsiveness to IL-4/IL-13 signaling from Th2 and ILC2 cells. Inhibition of IL-4/IL-13 signaling by dupilumab reduces expression of lipid peroxidation-associated genes, including *SLC27A2*. In eosinophilic CRSwNP, *SLC27A2* expression correlates with disease severity. Pharmacological inhibition of FATP2 in air–liquid interface cultures of nasal epithelial cells decreases expression of IL13RA1 and lipid peroxidation-related genes. Our findings identify FATP2-mediated lipid peroxidation as a key driver of epithelial dysfunction and inflammation in CRSwNP, providing new insights into disease mechanisms and potential therapeutic targets.

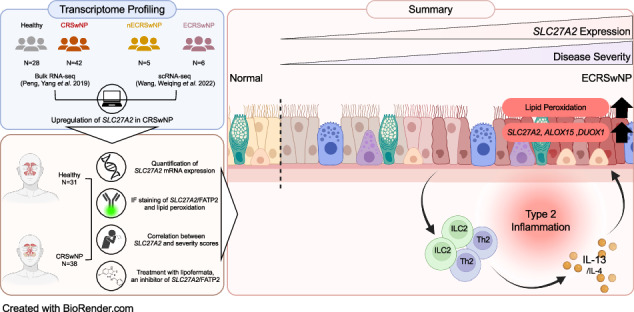

## Type 2 inflammation drives lipid peroxidation in nasal polyps

Chronic rhinosinusitis with nasal polyps affects many people, causing symptoms such as nasal congestion and loss of smell. Here the researchers found that type 2 inflammation drives molecular changes leading to epithelial dysfunction through lipid peroxidation in the nasal epithelium. A comprehensive study of patient tissue samples revealed the fatty acid transporter FATP2, encoded by the *SLC27A2* gene, in nasal polyp epithelium. IL-13 signaling from type 2 immune cells strongly induced SLC27A2 expression in epithelial cells. The researchers also discovered that pharmacological blockade of the IL-4/IL-13 pathway using dupilumab suppressed the expression of genes associated with lipid peroxidation. These findings suggest that targeting *SLC27A2* and related pathways could lead to new treatments for chronic rhinosinusitis with nasal polyps.

This summary was initially drafted using artificial intelligence, then revised and fact-checked by the author.

## Introduction

Chronic rhinosinusitis (CRS) is a prevalent disease affecting more than 10% of the population in Europe, South Korea and the USA, substantially impairing quality of life by presenting symptoms such as nasal congestion, discharge, facial pressure, anosmia, cough and fatigue^1–3^. CRS is traditionally classified into two distinct phenotypes: chronic rhinosinusitis with nasal polyps (CRSwNP) and chronic rhinosinusitis without nasal polyps (CRSsNP)^2^. The endotypes of CRSwNP are determined by the type of inflammation present, including eosinophilic CRSwNP (ECRSwNP) and non-eosinophilic CRSwNP (nECRSwNP)^3,4^. Recent evidence has elucidated the complex interplay between various inflammatory pathways and tissue remodeling factors, delineating their relevance to the endotypes of CRSwNP^5^. This growing understanding underscores the importance of a personalized, endotype-targeted approach to the management of CRSwNP^3,6^. Despite these advances, the selection of an optimal therapeutic strategy for CRSwNP remains challenging, particularly due to the lack of molecular biomarkers that adequately reflect the clinical characteristics of the various CRSwNP endotypes. Therefore, there is an urgent need to identify novel molecular markers that can guide the development of targeted therapies for patients with CRSwNP, considering the heterogeneous nature of the disease and its distinct endotypes.

To overcome the limitations in understanding the pathogenesis of CRSwNP, recent studies have focused on the genomic profiling of nasal polyps (NPs). Whole-transcriptome sequencing has uncovered gene signatures related to inflammation and abnormal host defense responses in CRSwNPs^7^. The differences in gene expression and molecular function between ECRSwNP and nECRSwNP have also been explored^8,9^. Single-cell transcriptome analysis of NP tissues showed an imbalanced epithelial cellular microenvironment, marked by hyperplasia of basal cells and ciliated cell loss^10,11^. *ALOX15*, *CCL26* and *CST1* have been identified as markers for promoting strong type 2 immunity^9–11^. Pro-inflammatory mediators, including *CCL13* and *CCL18* in ‘M2’-like macrophages and *IL-33* in conventional dendritic cells, have been detected in the immune cells of ECRSwNPs^9,12^. Thus, recent research has focused on studying the mechanisms underlying how inflammatory stimuli change immune cells and epithelial cells (EpCs) in NPs, serving as a repository for these chronic inflammatory memories^10^.

Metabolic dysregulation has been highlighted as a contributing factor to the pathogenesis of CRSwNP^11,13–17^. ALOX15, a lipid mediator involved in type 2 inflammation, has been identified in specific macrophage and dendritic cell subpopulations within NP tissue of ECRSwNP. ALOX15-positive macrophages exhibit elevated expression of type 2 chemokines (*CCL13*, *CCL18* and *CCL26*) as well as lipid transport genes (*PLTP,* *APOE*, *ABCA1* and *LRP1*)^11^. Dysregulated fatty acid metabolism has also been observed in NP-derived eosinophils^18^. Furthermore, imbalances in lipid metabolism can induce epithelial dysfunction; tuft cells within the NP epithelium uniquely overexpress genes related to the arachidonic acid metabolic pathway, such as *PTGS1* and *ALOX5 (*ref. ^14^). Interestingly, *ALOX15*, which is also highly expressed in the epithelium of NPs^19–21^, shows the expression of lipid peroxidation-related genes^22–24^. However, our understanding of the role of lipid metabolism in NP epithelial dysfunction remains incomplete.

In this study, we aimed to identify potential prognostic markers and therapeutic targets related to lipid metabolism in NP epithelial dysfunction in patients with CRSwNP. We performed an integrated analysis of bulk and single-cell RNA sequencing (scRNA-seq) data, which revealed upregulation of lipid metabolism-associated genes in EpCs and myeloid cells of NP tissue in CRSwNP compared with healthy nasal mucosa. Notably, the fatty acid transporter protein 2 (FATP2), encoded by solute carrier family 27-member 2 (*SLC27A2*), was significantly increased in NP EpCs, which coincided with lipid peroxidation staining. The *SLC27A2*-positive EpC subpopulation from patients with ECRSwNP preferentially interacts with type 2 immune cells, such as Th2 cells and ILC2, to receive IL-13 signals. Interestingly, we found a reduction in lipid peroxidation-related genes after treatment with dupilumab in patients with CRSwNP. Furthermore, *SLC27A2* expression positively correlated with disease severity scores in patients with ECRSwNP. Inhibition of FATP2 resulted in reduced expression of lipid peroxidation markers as well as the IL-13 receptor gene. These findings suggest that *SLC27A2* may serve as a potential prognostic marker and therapeutic target for patients with severe CRSwNP.

## Materials and methods

### Patient recruitment

We enrolled adult Korean participants with CRSwNPs and healthy controls from the Department of Otorhinolaryngology–Head and Neck Surgery at Asan Medical Center between July 2019 and December 2022. Written informed consent was obtained from all subjects before the study’s commencement, conducted in accordance with the Declaration of Helsinki. This study received approval from the Asan Medical Center Institutional Review Board (no. 2019-0619).

For patients with CRSwNPs, NP tissues were collected during functional endoscopic sinus surgery, and diagnoses followed the criteria outlined in the 2020 European Position Paper on Rhinosinusitis and Nasal Polyps guidelines, which included assessing symptoms, nasal endoscopy findings and computed tomography (CT) imaging^2^. Exclusion criteria included patients (1) younger than 18 years; (2) with a history of unilateral CRS, antrochoanal polyps, fungal sinusitis, allergic fungal sinusitis, aspirin-exacerbated respiratory disease, cystic fibrosis or primary ciliary dyskinesia; (3) who were pregnant or immunocompromised; (4) who had been administered decongestants, antibiotics and systemic/topical corticosteroids over 4 weeks preceding surgery; and (5) with a history of acute respiratory infection within 4 weeks before surgery.

Histopathological examination was performed to rule out fungal sinusitis, allergic fungal sinusitis, cystic fibrosis or primary ciliary dyskinesia. We also inquired about their history of acute upper and lower respiratory tract reactions to aspirin or nonsteroidal anti-inflammatory drugs to identify aspirin-exacerbated respiratory disease. Additionally, we conducted various assessments, including the Sinonasal Outcome Test (SNOT-22), Lund–Mackay CT scores, Lund–Kennedy endoscopic score, NP score and polyp grading according to the Davos classification^2,25,26^.

Control tissue samples were obtained from the middle turbinate mucosa of patients over 18 years old undergoing septoplasty for nasal obstruction without sinusitis on CT or transsphenoidal approach surgery for nonfunctioning pituitary tumors without adjacent structure invasion on magnetic resonance imaging and sinusitis on CT scan. These control subjects had no history of asthma, upper respiratory infection or administration of systemic, topical corticosteroids or antibiotic medications within 4 weeks before surgery. We determined atopic status by detecting specific IgE antibodies to common inhalant allergens using ImmunoCAP tests (Phadia), and asthma was diagnosed based on medical history and pulmonary function tests, including spirometry and challenge tests. The clinical characteristics of all subjects are presented in Table 1.

**Table 1:** Demographics and clinical characteristics of subjects.

	Control	CRSwNP
**IF staining**	**3**	**16**
Age (years), mean ± s.d.	54.3 ± 8.3	53.9 ± 12.5
Male, *n* (%)	2 (66.7)	14 (87.5)
Asthma, *n* (%)	0	3 (18.8)
Atopic, *n* (%)	0	5 (31.3)
SNOT-22, mean ± s.d.	8.7 ± 6.8	42.3 ± 21.1^‡^
Lund–Mackay CT score, mean ± s.d.	0	19.3 ± 4.3^‡^
Lund–Kennedy endoscopic score, mean ± s.d.	0	9.8 ± 2.8^‡^
NP score, mean ± s.d.	0	5.1 ± 2.4^‡^
Polyp grade, mean ± s.d.	0	3.8 ± 1.7^‡^
**RT–qPCR**	**20**	**31**
Age (years), mean ± s.d.	38.1 ± 15.4	50.4 ± 12.8^‡^
Male, *n* (%)	17 (85)	29 (93.5)
Asthma, *n* (%)	0	4 (12.9)^§^
Atopic, *n* (%)	13 (65)	14 (45.2)
SNOT-22, mean ± s.d.	39.5 ± 17.5	39.7 ± 20.2
Lund–Mackay CT score, mean ± s.d.	0.2 ± 0.5	18.1 ± 4.3^‡^
Lund–Kennedy endoscopic score, mean ± s.d.	0	10.1 ± 2.6^‡^
NP score, mean ± s.d.	0	5 ± 2.1^‡^
Polyp grade, mean ± s.d.	0	3.6 ± 1.4^‡^
**Oil Red O staining**	**3**	**7**
Age (years), mean ± s.d.	61.7 ± 17.2	53.6 ± 20.8
Male, *n* (%)	1 (33.3)	5 (71.4)
Asthma, *n* (%)	0	2 (28.6)
Atopic, *n* (%)	0	4 (57.1)
SNOT-22, mean ± s.d.	3.7 ± 4.7	38.9 ± 21.9^‡^
Lund–Mackay CT score, mean ± s.d.	0	16.9 ± 5.3^‡^
Lund–Kennedy endoscopic score, mean ± s.d.	0	9.7 ± 2.4^‡^
NP score, mean ± s.d.	0	3.7 ± 1.8^‡^
Polyp grade, mean ± s.d.	0	2.7 ± 0.9^‡^
**Lipofermata treatment**	**8**	**9**
Age (years), mean ± s.d.	47.1 ± 15.1	48.6 ± 10.8
Male, *n* (%)	4 (50)	6 (85.7)
Asthma, *n* (%)	0	0
Atopic, *n* (%)	3 (37.5)	2 (28.6)
SNOT-22, mean ± s.d.	9 ± 13	45.6 ± 24.4^‡^
Lund–Mackay CT score, mean ± s.d.	0	14.9 ± 4^‡^
Lund–Kennedy endoscopic score, mean ± s.d.	0	9.6 ± 2.8^‡^
NP score, mean ± s.d.	0	4 ± 1.6^‡^
Polyp grade, mean ± s.d.	0	3.1 ± 1.2^‡^

^‡^*P* < 0.05, Mann–Whitney *U* test.

^§^*P* < 0.05, chi-square test.

### Bulk RNA-seq data analysis

For the analysis of bulk RNA-seq data (GSE136825, GSE179269 and GSE268072), we mapped the reads to the hg38 genome using STAR version 2.7.3a with default parameters^27^. Subsequently, we converted the aligned reads into tag directories using HOMER version 4.11.1. Quantification of each file was performed utilizing the ‘analyzeRepeats’ script in HOMER. We normalized gene expression levels in each sample by calculating fragments per kilobase of transcript per million mapped reads (FPKM). Differentially expression genes (DEGs) were identified through DESeq2 analysis based on raw read counts^28^. The ‘getDifferentialExpression’ command in HOMER was applied with criteria for significance defined as an adjusted *P* value of <0.05, more than a twofold difference in expression levels and an average FPKM >2. Principal component analysis (PCA) was performed to assess sample clustering across two independent datasets: GSE136825 (28 healthy and 42 CRSwNP samples), GSE179269 (7 healthy and 17 CRSwNP samples) and GSE268072 (5 resting human nasal EpCs (hNECs) and 5 IL-4/IL-13 co-stimulated hNECs). In the GSE179269 dataset, one healthy sample clustered closely with the CRSwNP group and was omitted from subsequent analyses.

### ScRNA-seq analysis

#### Surgical biopsy of NP tissues scRNA-seq datasets

To profile the cellular ecosystem of NP tissues, we analyzed publicly available scRNA-seq data (accession number HRA000772)^11^. To ensure compatibility with the publicly available bulk RNA-seq dataset (GSE136825) and to maintain uniformity in disease states and tissue locations, we utilized data from 11 samples obtained from surgical biopsies of NP tissues. These samples encompassed five patients with nECRSwNP and six patients with ECRSwNP.

For our analysis, we included only cells in which mitochondrial RNA accounted for less than 15% of the total RNA content. We performed data integration and anchoring using the top 10,000 highly variable features identified through the variance-stabilizing transformation method. Subsequently, we conducted uniform manifold approximation and projection (UMAP) analysis and constructed a nearest-neighbor graph using the ‘FindNeighbors’ function. To identify cell clusters, we employed the ‘FindClusters’ function with a resolution parameter set to 0.5. Differential expression analysis was carried out using the ‘FindMarkers’ and ‘FindAllMarkers’ functions. Furthermore, we evaluated the expression of lipid metabolism (GO:0006629), reactive oxygen species (ROS) metabolism (GO:0072593) and inflammatory response (GO:0006954) genes across cell types using the ‘AddModuleScore’ function, which computes module scores based on associated gene ontology terms.

#### Nasal scrapings scRNA-seq datasets

To identify cell populations expressing elevated levels of *SLC27A2*, we utilized previously published scRNA-seq data^10^. These data included 7,886 cells from the ethmoid sinus of two patients with chronic rhinosinusitis with nasal polyps (CRSwNP-NP), 2,031 cells from the inferior turbinate (IT) of four patients with CRSwNP (CRSwNP-IT) and 6,498 cells from the IT of three healthy controls (Healthy-IT). We included only those cells in which mitochondrial RNA accounted for less than 20% of the total RNA content. Data integration and anchoring were performed using the top 3,500 highly variable features determined by the variance-stabilizing transformation method. Subsequently, we conducted UMAP analysis and constructed a nearest-neighbor graph using the ‘FindNeighbors’ function. Cluster identification was achieved using the ‘FindClusters’ function with a resolution parameter set to 0.5. DEGs were identified using the ‘FindMarkers’ and ‘FindAllMarkers’ functions.

#### Dupilumab treatment scRNA-seq datasets

To investigate single-cell transcriptomic changes upon IL-4/IL-13 signaling inhibition by dupilumab, we analyzed public scRNA-seq datasets^10^ of samples before and after dupilumab treatment. These datasets comprised 6,812 cells from two pretreatment patients with CRSwNP and 4,391 cells from three patients post-treatment. Only cells with less than 20% mitochondrial RNA content were included. Data integration and anchoring were performed using the top 1,500 highly variable features determined by a variance-stabilizing transformation. UMAP analysis was then conducted, and a nearest-neighbor graph was constructed using the ‘FindNeighbors’ function. Cluster identification employed the ‘FindClusters’ function with a resolution parameter of 0.5. DEGs were identified using the ‘FindMarkers’ and ‘FindAllMarkers’ functions.

#### Cell–cell communication analysis

Intercellular communication networks were delineated from scRNA-seq data using the CellChat version 2.1.1R package^29^. CellChat employs a curated database of ligand–receptor interactions, their cofactors and mass action kinetic models to quantify signaling communication patterns between cell types. This systematic approach identifies the dominant signaling pathways operative within a complex cellular milieu based on the expression patterns of ligands, receptors and cofactors across the constituent cell populations. Inference of cell type-specific signaling directionality is achieved through mathematical modeling of potential signaling configurations permitted by the measured gene expression profiles.

#### GO analysis

To identify enriched Gene Ontology (GO) terms among the DEGs, we employed Metascape^30^.

#### IF staining

For imunofluorescence (IF) staining, the samples (Table 1) were fixed in 4% paraformaldehyde in phosphate-buffered saline (PBS) and subsequently embedded in paraffin using standard procedures. Sections of the specimens, each 4 μm in thickness, were subjected to heat-induced antigen retrieval by boiling in 10 mM citrate buffer (pH 6.0) and allowed to cool to room temperature for 20 min. The specimens were then incubated overnight at 4 °C with anti-FATP2 (14048-1-AP, Proteintech; 1:100) antibodies. Afterward, the specimens were incubated with goat anti‐rabbit Alexa Fluor 488 (A11008, Invitrogen; 1:200) and BODIPY 581/591 C11 (D3861, Invitrogen; 10 μM) for 2 h at room temperature. Finally, the sections were counterstained with 4,6-diamidino-2-phenylindole (DAPI) (D1306, Invitrogen; 1:1,000) and examined using a confocal laser microscope (LSM 900, Carl Zeiss). Image analysis and quantification were performed using ImageJ version 1.53k.

#### ORO staining

To assess lipid droplets in the tissue, we employed Oil Red O (ORO) staining. Fixed-frozen tissue samples (Table 1) were rinsed with 60% isopropanol for 2 min, followed by staining with a freshly prepared ORO working solution (Sigma-Aldrich, O1391) for 15 min. After staining, the sections were rinsed three times with 60% isopropanol (1 min each) and twice with distilled water. Subsequently, sections were counterstained with 30% modified Mayer’s hematoxylin for 30 s, rinsed with tap water and two changes of distilled water and finally mounted in aqueous mounting media (Aquatex, Sigma-Aldrich).

#### RT–qPCR

Total RNA was extracted from nasal epithelia cells of CRSwNPs and healthy controls (Table 1) using the RNeasy Mini kit (Qiagen). Reverse transcription was carried out using 500 ng of total RNA with the RevertAid First Strand cDNA Synthesis kit (Thermo Fisher Scientific). Quantitative PCR with reverse transcription (RT–qPCR) was performed with TOPreal qPCR 2× PreMIX (SYBR Green with low ROX; RT500M, Enzynomics Co. Ltd.). The qPCR conditions included an initial cycle at 95 °C for 10 min, followed by 50 cycles of 10 s at 95 °C for denaturation, 15 s at 60 °C for annealing and 20 s at 72 °C for extension. A melting program was executed at 72–95 °C with a heating rate of 1 °C/45 s. The Rotor-Gene Q v2.3.1 (Qiagen) was utilized to capture and analyze spectral data. The sequences of primers used to assess mRNA expression are provided in Supplementary Table 1.

#### ALI culture

Primary hNECs were isolated from either polyp or control tissues (Table 1). Passage-2 hNECs (1.5 × 10^5^ cells/well) were seeded in 0.25 ml of culture medium on Transwell clear culture inserts (12 mm, with a 0.4 μm pore size; Costar, Corning Inc.). Initially, cells were cultured in a 1:1 mixture of basal epithelial growth medium and Dulbecco’s modified Eagle’s medium containing previously described supplements in a submerged state for the first 7–9 days^31,32^. Upon reaching confluence, the apical medium was removed to establish an air–liquid interface (ALI), and thereafter, the medium was replenished only in the basal compartment. RNA was extracted from hNECs on day 14 following ALI establishment^31,32^.

#### In vitro lipofermata treatment

At day 14 of ALI culture, hNECs were exposed to either dimethyl sulfoxide (DMSO; Sigma, 276855-100 ML) or 2 μM lipofermata (MedChemExpress, HY-116788), a FATP2 inhibitor, in the apical compartment for 24 h. RNA was subsequently extracted from HNECs.

#### Utilization of publicly available data

In this study, we employed various publicly available datasets and databases as follows (Table 2): Bulk RNA-seq datasets from GSE136825 (ref. ^7^), GSE179269 (ref. ^9^) and GSE268072 (ref. ^33^) and scRNA-seq datasets from HRA000772 (ref. ^11^) and ref. ^10^.

**Table 2:** Utilization of publicly available datasets.

Figure (main)	Figure (sub)	Dataset type	Accession number/reference	Samples	Source
Figure 1a–f Figure 2c Figure 3d Figure 4a, b Figure 7c, d	Figure 1a–d Figure 7c	Bulk RNA-seq	GSE136825	28 Healthy 42 CRSwNP	GEO Database
Figure 4a, b	Figure 3a, b Figure 7a–c	Bulk RNA-seq	GSE179269	6 Healthy 17 CRSwNP	GEO Database
Figure 6e, f	–	Bulk RNA-seq	GSE268072	5 Resting hNECs 5 IL-4/IL-13 co-stim. hNECs	GEO Database
Figure 2a–e Figure 3c, e Figure 5b–f Figure 6a–d	Figure 2a, b Figure 3d Figure 5a–f Figure 6a–e	scRNA-seq	HRA000772	11 CRSwNP	GSA-Human National Genomics Data Center (NGDC)
Figure 4f	–	scRNA-seq	Ref. ^10^	2 CRSwNP-NP 4 CRSwNP-IT 3 Healthy-IT	The Alexandria Project
Figure 6h–j	Figure 6f	scRNA-seq	Ref. ^10^	2 Dupilumab pretreatment 3 Dupilumab post-treatment	The Alexandria Project

### Statistical analysis

Statistical analyses were performed using GraphPad Prism version 10 (GraphPad Software). Data are presented as mean ± s.d., unless otherwise specified in figure legends. Statistical significance was determined using two-tailed Welch’s *t*-test, multiple unpaired Welch’s *t*-tests, Wilcoxon signed-rank test or paired *t*-test as appropriate. For all analyses, similar expected variances were assumed between compared groups. Statistical significance was set at *P* < 0.05, with *P < 0.05, **P < 0.01, ***P < 0.001 and ****P < 0.0001. The specific test used and exact *P* values are indicated in figure legends.

## Results

### Transcriptomic profiling reveals a lipid metabolic signature in NP tissues

**Fig. 1:**
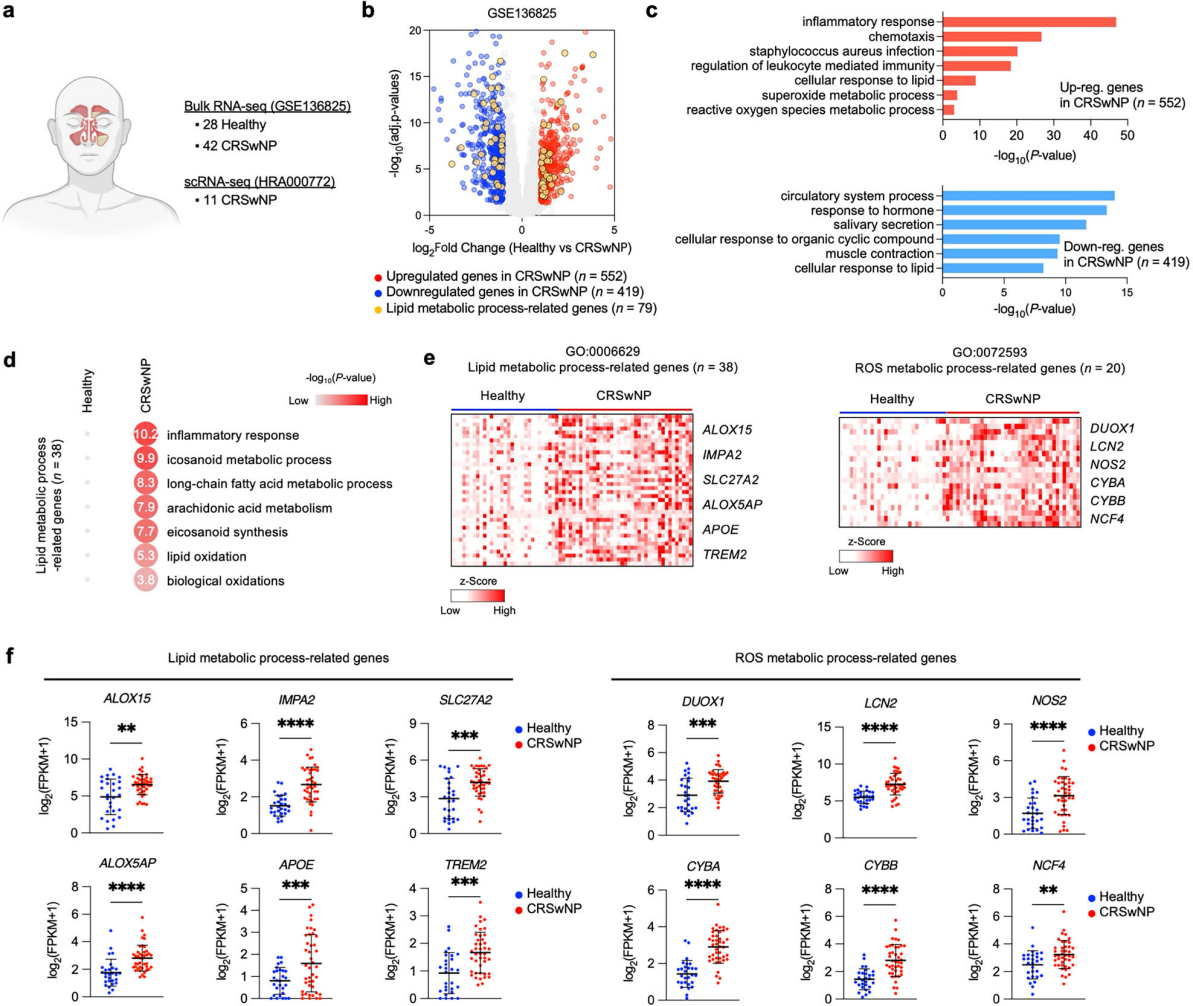
Dysregulated expression of lipid metabolism-related genes in NP tissues. **a** Integrative transcriptomic profiling utilizing datasets GSE136825 and HRA000772. The schematic depicts the integrated analysis approach combining bulk RNA-seq dataset GSE136825, comparing IT tissues from healthy controls (*n* = 28) to NP tissues from CRSwNP (*n* = 42), and the scRNA-seq dataset HRA000772, differentiating NP tissues from ECRSwNP (*n* = 6) and nECRSwNP (*n* = 5). Diagrams created using BioRender (https://biorender.com/). **b** Volcano plot demonstrating the transcriptomic expression differences between healthy and CRSwNP in the GSE136825 dataset. Genes with a significant false discovery rate <0.05 and a >2-fold difference in expression level with an average FPKM >2 are represented by colored dots (red, upregulated genes in CRSwNP; blue, downregulated genes in CRSwNP; yellow, lipid metabolic process-related genes). **c** GO enrichment analysis of 552 upregulated (top) and 419 downregulated (bottom) genes in CRSwNP versus healthy controls using Metascape. **d** GO analysis of the 38 upregulated genes associated with lipid metabolic processes from the 552 upregulated genes in CRSwNP. **e** Heat maps showing upregulated genes in CRSwNP involved in lipid metabolic processes (left) and ROS metabolic processes (right). Values are *z* scores. **f** Expression levels of representative genes associated with lipid metabolic processes (left) and ROS metabolic processes (right) in healthy controls and patients with CRSwNP. The error bars represent mean values ± s.d. *P* values determined by Welch’s *t*-test. *P < 0.05, **P < 0.01, ***P < 0.001 and ****P < 0.0001.

To elucidate unique molecular signatures within distinct cell types of NP tissues from patients with CRSwNP, we employed a comprehensive transcriptomic approach utilizing bulk RNA-seq (GSE136825; 28 healthy and 42 CRSwNP)^7^ and scRNA-seq datasets (HRA000772; 11 CRSwNP)^11^ **(**Fig. 1a). Using the bulk RNA-seq dataset, we identified a total of 971 DEGs, with 552 upregulated and 419 downregulated genes in CRSwNP (Fig. 1b). GO analysis of the DEGs revealed distinct molecular mechanisms and biological functions associated with upregulated and downregulated genes in CRSwNP. The GO terms ‘inflammatory response’, ‘cellular response to lipid’ and ‘superoxide metabolic process’ were highly enriched in upregulated genes (Fig. 1c, top), while ‘circulatory system process’, ‘response to hormone’, ‘salivary secretion’ and ‘muscle contraction’ were associated with downregulated genes **(**Fig. 1c, bottom). For the ‘inflammatory response’ category, 81 genes were found to be upregulated in CRSwNP samples relative to healthy controls (Supplementary Fig. 1a). Notable examples included *C3*, *IL20RB*, *SAA2*, *AIF1*, *CSF1R* and *HMOX1*, which exhibited markedly higher mRNA levels in NP tissues (Supplementary Fig. 1b).

Interestingly, the ‘cellular response to lipid’ GO term was found in both upregulated and downregulated genes, but the specific lipid metabolic pathways showed distinct functions. The upregulated lipid metabolic genes were enriched in functions related to ‘icosanoid metabolic process’, ‘arachidonic acid metabolism’ and ‘lipid oxidation’ (Fig. 1d). Genes associated with these pathways, such as *ALOX15*, *IMPA2*, *SLC27A2*, *ALOX5AP*, *APOE* and *TREM2* exhibited significantly elevated expression levels in patients with CRSwNP compared with healthy controls. Additionally, genes involved in ROS metabolic pathways, including *DUOX1*, *LCN2*, *NOS2*, *CYBA*, *CYBB* and *NCF4* were also upregulated in the disease state (Fig. 1e, f and the gene list is presented in Supplementary Table 2). In contrast, the downregulated genes associated with lipid metabolism displayed functions related to ‘cholesterol homeostasis’, ‘sterol homeostasis’ and ‘lipid homeostasis’ (Supplementary Fig. 1c). Genes involved in these pathways, such as *FABP4*, *INSIG1* and *LDLR*, displayed decreased expression levels in CRSwNP samples compared with healthy controls (Supplementary Fig. 1d). These results demonstrate that transcriptomic analysis reveals distinctive molecular signatures in CRSwNP, characterized by upregulated inflammatory and lipid metabolic pathways, particularly highlighting dysregulated gene expressions in icosanoid metabolism, arachidonic acid metabolism and ROS-associated processes.

### Single-cell transcriptional profiling reveals dysregulated lipid metabolism signatures in NP EpCs

**Fig. 2:**
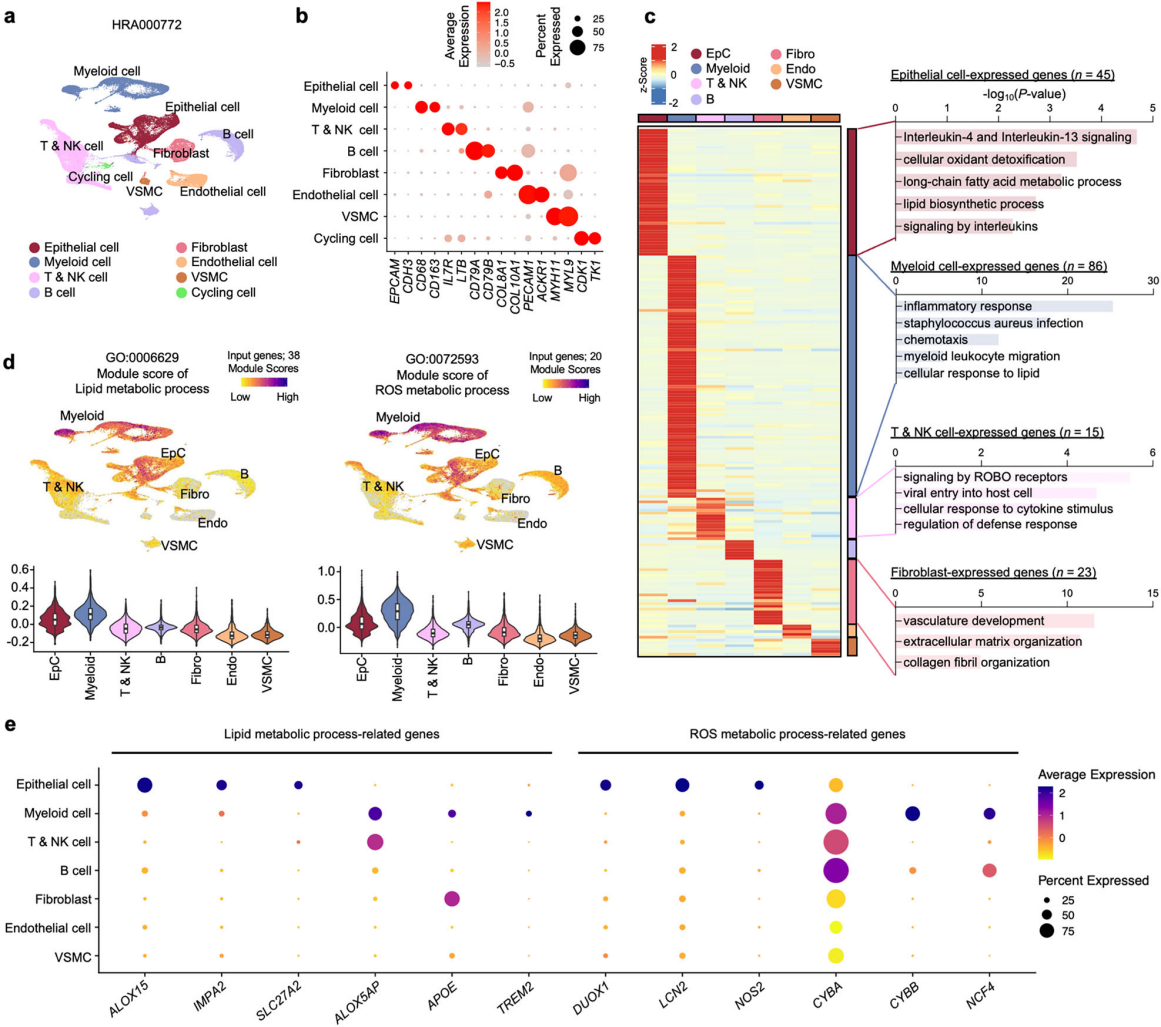
Single-cell landscape of dysregulated lipid metabolism-related genes in NPs. **a** UMAP of the public scRNA-seq dataset (HRA000772) for 72,032 single cells, where points are colored by cell identity. **b** A dot plot of representative gene expression levels mapped onto each cell type. **c** A heat map displaying the expression patterns of cell type-specific gene sets among upregulated DEGs in NP tissues, with values represented as *z* scores (left). GO annotations for the marker gene sets associated with each distinct cell type (right). **d** UMAP (top) and violin plots (bottom) showing the single-cell module scores for lipid metabolic processes (left) and ROS metabolic processes (right) across different cell types in NP tissues. **e** Dot plots depicting the expression patterns of representative genes associated with lipid metabolic processes and ROS metabolic processes in different cell types from CRSwNP-NP tissues. Each dot represents the percentage of cells expressing the given gene within each cell type, and the color scale indicates the average expression level. endo endothelial cell, fibro fibroblast, ROBO roundabout.

To further dissect the cellular composition and cell type-specific contributions to the observed transcriptional changes in NP tissues, we performed single-cell-level profiling using UMAP on the HRA000772 dataset. We identified eight distinct cell clusters encompassing 72,032 cells, including EpCs, myeloid cells, B cells, T and natural killer (NK) cells, fibroblasts, endothelial cells, vascular smooth muscle cells (VSMC) and cycling cells (Fig. 2a). Cluster-specific marker genes were used to delineate these cell types (Fig. 2b and the gene list is presented in Supplementary Table 3). Excluding the cycling cell cluster, we proceeded with subsequent analyses focusing on the seven primary cell types to ensure a robust cell type-specific characterization of the NP tissue transcriptional landscape. By integrating bulk and scRNA-seq data through deconvolution analysis, we identified genes predominantly expressed in each cell type, with a particular focus on those upregulated in patients with CRSwNP (Fig. 2c). These included 45 genes in EpCs, 86 in myeloid cells, 15 in T and NK cells, 7 in B cells, 23 in fibroblasts, 5 in endothelial cells and 6 in VSMC (Fig. 2c, left, and the gene list is presented in Supplementary Table 4). For the key categories mentioned above, GO terms related to lipid and fatty acid metabolic processes were predominantly enriched in EpC and myeloid cell clusters. While genes associated with inflammatory response were highly enriched in myeloid cells, EpCs showed notable enrichment of genes linked to type 2 inflammation, particularly those involved in the ‘interleukin-4 (IL-4) and interleukin-13 (IL-13) signaling’ pathways (Fig. 2c, right). Moreover, EpC and myeloid cell clusters exhibited relatively higher module scores for genes associated with lipid metabolism, ROS metabolism and inflammatory response compared with other cell types (Fig. 2d, Supplementary Fig. 2a and the gene list is presented in Supplementary Table 2). Focusing on the EpC and myeloid cell populations, representative genes from each GO category exhibited the highest expression levels in their respective clusters (Fig. 2e, b). Taken together, these suggest that distinct lipid metabolic signature genes are significantly upregulated in nasal EpCs within NP tissue, implying a role for altered lipid metabolism in the dysregulation of EpCs in patients with CRSwNP.

### Enhanced lipid peroxidation in the NP epithelium of patients with CRSwNP

**Fig. 3:**
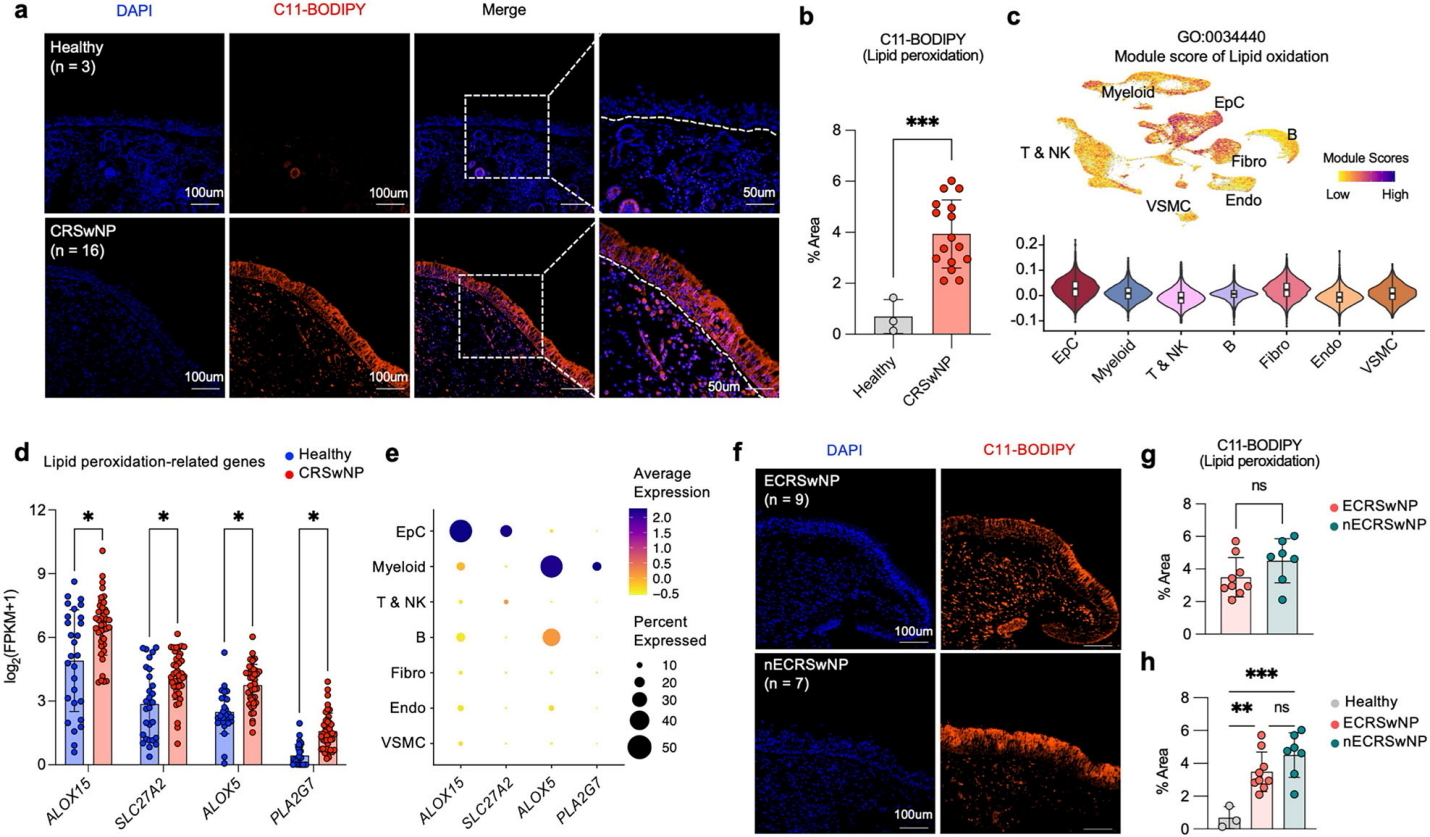
Enhanced lipid peroxidation in NP epithelium of patients with CRSwNP. **a** Representative IF staining images of C11-BODIPY (red) co-localized with DAPI (blue) in both healthy (*n* = 3) and CRSwNP samples (*n* = 16). **b** A bar graph showing the percentage area of C11-BODIPY staining, a marker of lipid oxidation, quantified from tissue sections of healthy controls and CRSwNP. **c** UMAP (top) and violin plots (bottom) showing the single-cell module scores for lipid oxidation across different cell types in CRSwNP NP tissues. **d** The graph depicts mRNA expression levels of four representative genes involved in lipid oxidation processes in NP tissues from healthy controls and patients with CRSwNP. *P* values determined by multiple unpaired Welch’s *t*-tests for each gene. *Adjusted *P* < 0.05. **e** A dot plot showing *ALOX15*, *SLC27A2*, *ALOX5* and *PLA2G7* expression levels across onto each cell type. **f** Representative IF staining images of C11-BODIPY (red) co-localized with DAPI (blue) in both ECRSwNP (*n* = 9) and nECRSwNP samples (*n* = 7). **g** A bar graph showing the percentage area of C11-BODIPY staining, a marker of lipid oxidation, quantified from tissue sections of ECRSwNP and nECRSwNP. **h** A bar graph showing the percentage area of C11-BODIPY staining quantified in tissue sections from healthy, ECRSwNP and nECRSwNP groups. Scale bars, 50 μm and 100 μm. Error bars represent mean values ± s.d. *P* values determined by multiple unpaired Welch’s *t*-tests (**d**), Welch’s *t*-test (**b** and **g**) and Welch’s analysis of variance (**h**). *P < 0.05, **P < 0.01, ***P < 0.001 and ****P < 0.0001.

Our transcriptomic analysis revealed an upregulation of genes associated with lipid oxidation in NP tissues from patients with CRSwNP. To validate these findings at the protein level, we compared lipid peroxidation levels between NP samples from patients with CRSwNP and healthy controls using C11-BODIPY, a ratiometric fluorescent sensor. C11-BODIPY staining of tissue section (3 healthy and 16 CRSwNP) revealed markedly stronger fluorescence levels in NP tissues, with particularly strong signals in the epithelial layer of NP tissues (Fig. 3a, b). At the single-cell level, the EpC cluster exhibited the highest module score for the lipid oxidation gene signature (Fig. 3c and the gene list is presented in Supplementary Table 2), which includes *ALOX15*, *SLC27A2*, *ALOX5* and *PLA2G7*, genes that are significantly upregulated in CRSwNP compared with healthy controls (Fig. 3d). Among these genes, *ALOX15* and *SLC27A2* exhibited pronounced expression in the EpC population (Fig. 3e), suggesting their potential role in driving lipid oxidation processes within the NP epithelium. To further validate our findings, we analyzed an independent bulk RNA-seq dataset (GSE179269; 6 healthy and 17 CRSwNP) from another cohort. Consistent with our initial results, the lipid mediator *ALOX15* and fatty acid transporter *SLC27A2* were significantly upregulated in patients with CRSwNP compared with healthy controls (Supplementary Fig. 3a). Additionally, genes involved in ROS metabolism, such as *DUOX1* and *NOS2*, were also significantly increased in CRSwNP (Supplementary Fig. 3b), further supporting the presence of enhanced oxidative stress in NP tissues.

Next, we investigated whether lipid peroxidation signatures in the nasal epithelium differed between the two main endotypes of CRSwNP:ECRSwNP and nECRSwNP (Supplementary Fig. 3c). Interestingly, C11-BODIPY staining intensity revealed no significant differences between ECRSwNP (*n* = 9) and nECRSwNP (*n* = 7) endotypes, but showed substantial differences in both endotypes compared with healthy controls (*n* = 3) (Fig. 3f–h). Furthermore, the module scores for the lipid oxidation gene signature at the single-cell level were comparable between the two endotypes (Supplementary Fig. 3d), suggesting that enhanced lipid peroxidation is a common feature of CRSwNP, regardless of the endotype. These results demonstrate that lipid peroxidation is markedly increased in the NP epithelium in patients with CRSwNP, associated with the upregulation of lipid oxidation-related genes such as *ALOX15* and *SLC27A2* in the EpC population. This enhanced lipid peroxidation signature appears to be a shared characteristic of both eosinophilic and non-eosinophilic CRSwNP endotypes.

### FATP2 overexpression in NP epithelium of patients with CRSwNP

**Fig. 4:**
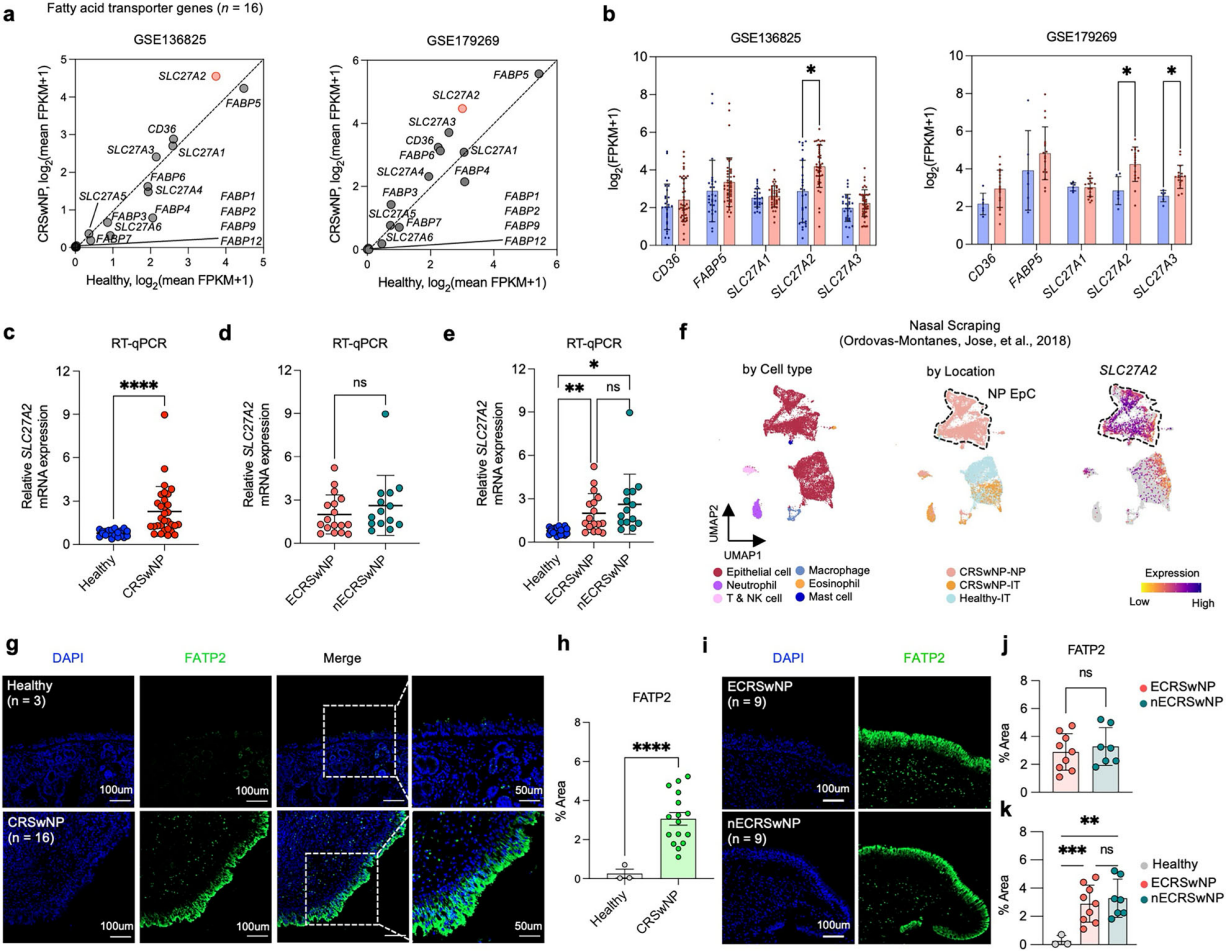
Elevated *SLC27A2*/FATP2 expression in NP epithelium. **a** A scatter plot showing the disparities in gene expression levels of fatty acid transporter-encoding genes between the CRSwNP and healthy groups based on the GSE136825 and GSE179269 dataset. **b** Comparing the expression levels of fatty acid transporter-encoding genes in CRSwNP compared with healthy groups, drawing on data from GSE136825 (left) and GSE179269 (right). Genes with mean log_2_(FPKM + 1) values lower than 2 were excluded. **c** mRNA expression levels of *SLC27A2*, quantified via RT–qPCR and normalized to GAPDH mRNA, comparing CRSwNP (*n* = 31) and healthy (*n* = 20) groups. **d** mRNA expression levels of *SLC27A2*, quantified via RT–qPCR and normalized to GAPDH mRNA, comparing ECRSwNP (*n* = 17) and nECRSwNP (*n* = 14). **e** mRNA expression levels of *SLC27A2*, quantified via RT–qPCR and normalized to GAPDH mRNA, comparing healthy (*n* = 20), ECRSwNP (*n* = 17) and nECRSwNP (*n* = 14). **f** Single-cell transcriptomic analysis of nasal cells from healthy and CRSwNP subjects (nasal scraping data from ref. ^10^). UMAP projection of 16,415 single cells from nasal scrapings, with cells colored by assigned cell type annotations (left), sample origin (CRSwNP ethmoid sinus polyp (7,886 cells, *n* = 2 CRSwNP-NP), CRSwNP-IT (2,031 cells, *n* = 4 CRSwNP-IT) or healthy control IT (6,498 cells, *n* = 3 Healthy-IT) (middle)) and expression of *SLC27A2* (right). **g** IF staining images of FATP2 (green) combined with DAPI (blue) for healthy (*n* = 3) and CRSwNP samples (*n* = 16). **h** A bar graph depicting the percentage area of FATP2 staining quantified from tissue sections of healthy controls and CRSwNP samples. **i** IF staining images of FATP2 (green) and DAPI (blue) for NP tissue sections from ECRSwNP (*n* = 9) and nECRSwNP (*n* = 7) groups. **j** A bar graph showing the percentage area of FATP2 staining quantified from tissue sections of ECRSwNP and nECRSwNP groups. **k** A bar graph showing the percentage area of FATP2 staining quantified in tissue sections from healthy, ECRSwNP and nECRSwNP groups. Scale bars, 50 μm and 100 μm. The error bars represent mean values ± s.d. *P* values determined by multiple unpaired Welch’s *t*-tests (**b**), Welch’s *t*-test (**c**, **d**, **h** and **j**) and Welch’s analysis of variance (**e** and **k**). *P < 0.05, **P < 0.01, ***P < 0.001 and ****P < 0.0001.

Previous studies have revealed that fatty acid transporters play a critical role in regulating lipid metabolism, which is linked to lipid homeostasis in various cellular abnormalities and diseases^34–37^. To investigate their potential involvement in CRSwNP, we evaluated the gene expression levels of all known fatty acid transporters, including the solute carrier 27A (SLC27A) gene family, fatty acid binding proteins (FABPs) and CD36 in bulk RNA-seq datasets from two independent cohorts (GSE136825 and GSE179269) (Fig. 4a). Of the 16 fatty acid transporter genes analyzed, 11 genes were excluded due to low expression levels. Among the remaining five genes, only *SLC27A2* exhibited a pronounced elevation in gene expression in CRSwNP compared with healthy controls (Fig. 4b). To validate the upregulation of *SLC27A2* in CRSwNP, we performed RT–qPCR experiments on nasal tissue samples. Consistent with the RNA-seq data, *SLC27A2* mRNA expression was significantly higher in the NP tissue of patients with CRSwNP compared with healthy controls (Fig. 4c). However, when comparing the differences between the two main endotypes (Supplementary Fig. 4a), the differences were not significant and there was a significant increase in each endotype relative to the healthy control (Fig. 4d, e).

To pinpoint the localization of *SLC27A2* expression, we utilized a public scRNA-seq dataset of nasal scrapings from healthy IT (Healthy-IT; *n* = 3), as well as IT (CRSwNP-IT; *n* = 4) and NP (CRSwNP-NP; *n* = 2) from patients with CRSwNP^10^. Our analysis revealed that *SLC27A2* exhibited the highest expression levels in EpCs of NP tissues compared with IT tissues from both healthy controls and patients with CRSwNP (Fig. 4f). This finding suggests that the upregulation of *SLC27A2* in CRSwNP is primarily driven by its increased expression in the EpCs of NPs.

FATP2, the protein encoded by *SLC27A2*, is a marker of lipid peroxidation as well as lipid accumulation^36,38,39^. To confirm the presence of lipid accumulation and localize FATP2 expression in the nasal epithelium, we performed ORO staining and anti-FATP2 IF staining on nasal tissue sections from patients with CRSwNP and healthy controls. ORO staining revealed increased lipid accumulation in the epithelium of NPs compared with healthy controls (Supplementary Fig. 4b). Consistent with this finding, anti-FATP2 IF staining demonstrated significantly elevated FATP2 levels in the epithelium of NPs compared with healthy controls (Fig. 4g, h), suggesting that the increased expression of FATP2 in the NP epithelium may contribute to the observed lipid accumulation in CRSwNP. However, FATP2 levels did not differ significantly between ECRSwNP and nECRSwNP, which is consistent with the results of lipid peroxidation intensities and *SLC27A2* mRNA expression (Fig. 4i–k). Collectively, these data reveal that *SLC27A2*/FATP2 is significantly overexpressed in the NP epithelium of patients with CRSwNP compared with healthy controls, irrespective of endotype. The increased expression of *SLC27A2*/FATP2 in the NP epithelium may contribute to the enhanced lipid peroxidation and accumulation observed in CRSwNP, highlighting its potential role in the pathogenesis of this disease.

### Elevated lipid peroxidation and distinct gene signatures in *SLC27A2*^+^ NP EpCs of CRSwNP

**Fig. 5:**
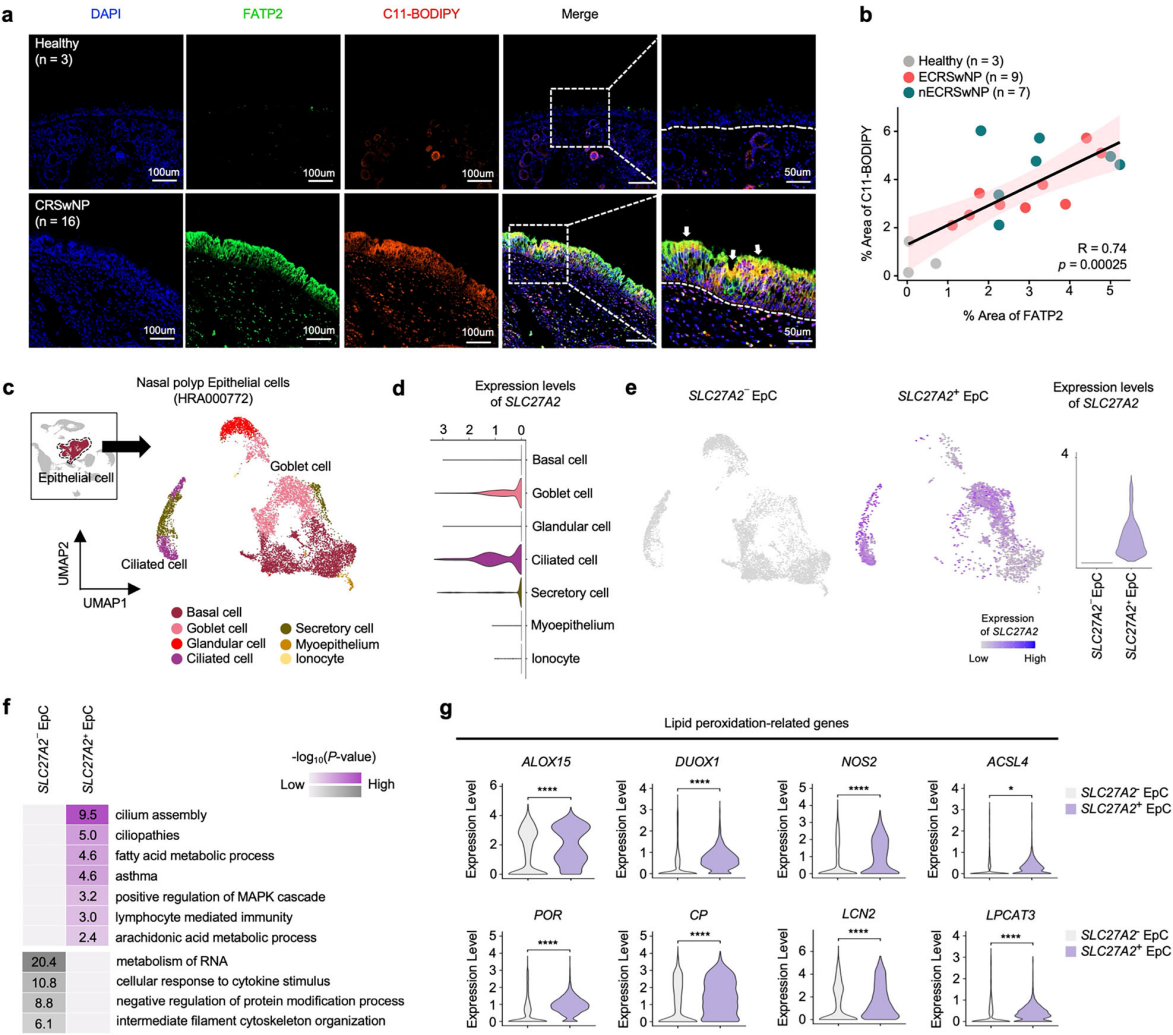
Transcriptomic signatures and elevated lipid peroxidation in *SLC27A2*^+^ NP EpCs. **a** IF staining of FATP2 (green) and C11-BODIPY (red) merged with DAPI (blue) in healthy tissue (*n* = 3) and CRSwNP tissue (*n* = 16). **b** A scatter plot depicting correlations between the percentage area stained by anti-FATP2 and C11-BODIPY. Spearman correlation coefficients (*R*) and associated *P* values are depicted. **c** UMAP plot displaying 9,148 EpCs from 11 patients with CRSwNP categorized into 7 subsets. **d** A violin plot illustrating the expression levels of *SLC27A2* across seven EpC subsets. **e** UMAP plots (left) depicting *SLC27A2*^−^ and *SLC27A2*^+^ EpC in NPs from 11 patients with CRSwNP. *SLC27A2* expression is color indicated. A violin plot (right) showing the expression levels of *SLC27A2* in *SLC27A2*^−^ and *SLC27A2*^+^ EpC. **f** A heat map representing the enriched GO terms of DEGs between *SLC27A2*^−^ and *SLC27A2*^+^ EpCs. Values are −log_10_(*P* value). **g** A violin plot displaying the expression levels of lipid peroxidation-related genes in *SLC27A2*^−^ and *SLC27A2*^+^ EpCs. Scale bars, 50 μm and 100 μm. *P* values were derived from Wilcoxon signed-rank tests. *P < 0.05, **P < 0.01, ***P < 0.001 and ****P < 0.0001.

To investigate the relationship between lipid peroxidation and FATP2, we performed IF staining on NP tissue sections. Our analysis revealed co-localization of FATP2 and lipid peroxidation markers in the NP epithelium (Fig. 5a), suggesting a potential link between FATP2 expression and lipid peroxidation in this tissue. Furthermore, we quantified the FATP2 coverage area and the extent of lipid peroxidation in the NP epithelium and found a direct correlation between these two parameters (Fig. 5b).

To gain insights into the transcriptomic profiles of *SLC27A2*-expressing EpCs within the NP epithelium, we analyzed scRNA-seq data. From the heterogeneous cell populations in NP tissues, we isolated the EpC cluster and subdivided it into seven subclusters (Fig. 5c), each characterized by specific epithelial subpopulation marker genes (Supplementary Fig. 5a). *SLC27A2* expression was predominantly localized to ciliated and goblet cell subclusters, with the highest expression levels observed in ciliated cells (Fig. 5d). By applying an *SLC27A2* expression cutoff of 0, we categorized EpCs into *SLC27A2*-negative (*SLC27A2*^−^ EpC) and *SLC27A2*-positive (*SLC27A2*^+^ EpC) populations for comparative analyses (Fig. 5e). *SLC27A2*^+^ EpCs constituted 30.18% of the NP epithelium (Supplementary Fig. 5b) and showed a marked presence in goblet cells (43.10%) and ciliated cells (13.76%) compared with *SLC27A2*^−^ EpCs (goblet cells, 19.24% and ciliated cells, 5.06%) (Supplementary Fig. 5c). GO analysis revealed that *SLC27A2*^+^ EpCs were enriched in genes associated with ‘ciliopathies’, ‘fatty acid metabolism’, ‘lymphocyte-mediated immunity’ and ‘arachidonic acid metabolic process’ pathways. In contrast, *SLC27A2*^−^ EpCs showed an enrichment of genes involved in ‘RNA metabolism’, ‘cytokine response signaling’ and ‘negative regulation of protein modifications’ (Fig. 5f). Notably, lipid peroxidation-related genes, including *ALOX15*, *DUOX1*, *NOS2*, *ACSL4*, *POR*, *CP*, *LCN2* and *LPCAT3*, were significantly upregulated in *SLC27A2*^+^ EpC compared with *SLC27A2*^−^ EpC (Fig. 5g), further highlighting the association between *SLC27A2* expression and lipid peroxidation.

Additionally, we investigated the transcriptomic signature of *SLC27A2*^+^ EpC clusters between ECRSwNP and nECRSwNP populations (Supplementary Fig. 5d). As expected, the IL-4 and IL-13 signaling GO term was highly enriched in *SLC27A2*^+^ EpCs of ECRSwNP, while type I inflammation and Th17-related GO terms were preferentially enriched in *SLC27A2*^+^ EpCs of nECRSwNP (Supplementary Fig. 5e,f). Interestingly, reactive oxygen-related pathways and lipid metabolism genes were enriched in *SLC27A2*^+^ EpCs of both ECRSwNP and nECRSwNP (Supplementary Fig. 5e,f), suggesting an imbalance in lipid and fatty acid metabolism and increased lipid peroxidation in *SLC27A2*^+^ EpCs in both endotypes. Our findings suggest that *SLC27A2* expression is primarily localized in goblet and ciliated cell types and that *SLC27A2*^+^ EpCs may be associated with imbalanced lipid and fatty acid metabolism, ciliopathies and inflammatory responses. Moreover, the significantly higher levels of lipid peroxidation markers observed in *SLC27A2*^+^ EpCs indicate a link between *SLC27A2*/FATP2 and impaired lipid homeostasis, leading to lipid oxidative injury in NPs.

### Interaction between *SLC27A2*^+^ EpCs and immune cells via IL-13 signaling in ECRSwNP

**Fig. 6:**
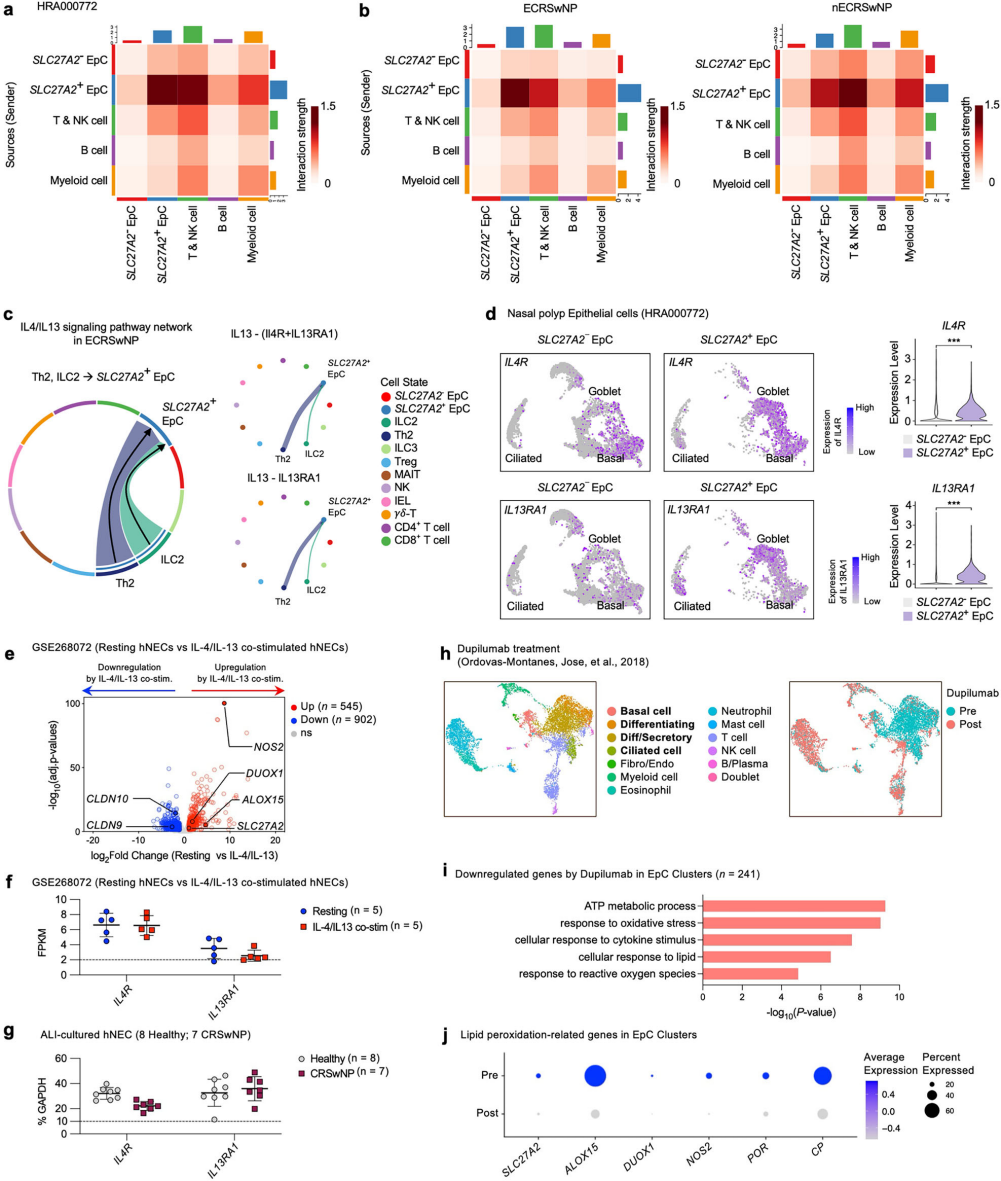
Interplay between *SLC27A2*^+^ EpCs and Th2/ILC2 cells through the IL-13 signaling pathway in ECRSwNP. **a** A heat map depicting cell–cell interaction strengths between immune cell types with *SLC27A2*^−^ and *SLC27A2*^+^ EpC. **b** Heat maps illustrating the interaction profiles between immune cell types with *SLC27A2*^−^ and *SLC27A2*^+^ EpC and in eosinophilic (left) and non-eosinophilic CRSwNP (right). The color intensity represents interaction strength (scale 0–1.5), with darker red indicating stronger interactions. **c** Chord diagrams illustrating the predicted IL-4/IL-13 signaling from EpC and T and NK cell types in ECRSwNP (left). Circular plots depicting the contribution of each cell type to the overall IL-13 signaling in ECRSwNP (right). The thickness of the lines corresponds to the signal intensity, with thicker lines indicating stronger signaling. **d** UMAP plots (left) depicting *SLC27A2*^−^ and *SLC27A2*^+^ EpC in NPs. *IL4R* and *IL13RA1* expression is color indicated. A violin plot (right) showing the expression levels of *IL4R* and *IL13RA1* in *SLC27A2*^−^ and *SLC27A2*^+^ EpC. **e** A volcano plot demonstrating the transcriptomic expression differences between resting hNECs and IL-4/IL-13 co-stimulated hNECs (GSE268072, resting hNECs *n* = 5 and IL-4/IL-13 co-stimulated hNECs *n* = 5). Genes with a significant false discovery rate <0.05 and a >2-fold difference in expression level with an average FPKM >2 are represented by colored dots (red, upregulated gene in IL-4/IL-13 co-stimulated hNECs; blue, downregulated gene in IL-4/IL-13 co-stimulated hNECs). **f** FPKM values of *IL4R* and *IL13RA1* in ALI-cultured hNECs under resting conditions and IL-4/IL-13 co-stimulation (*n* = 5 per group). **g** mRNA expression levels of *IL4R* and *IL13RA1*, quantified via RT–qPCR and normalized to GAPDH mRNA, in ALI-cultured hNEC in healthy individuals (*n* = 8) and patients with CRSwNP (*n* = 7). **h** Single-cell transcriptomic profiles of nasal cells from patients with CRSwNP before and after dupilumab treatment (dupilumab data from ref. ^10^; pretreatment *n* = 2 and post-treatment *n* = 3 patients). UMAP of 11,203 single-cell transcriptomes colored by cell type annotations (left) and dupilumab treatment status (right). **i** GO analysis of the 241 downregulated genes by dupilumab in EpC clusters, performed using Metascape. **j** A dot plot showing the expression changes of genes associated with lipid peroxidation before and after treatment with dupilumab. *P* values were derived from a Wilcoxon signed-rank test (**d**). * *P* < 0.05, **P < 0.01, ***P < 0.001 and ****P < 0.0001.

To explore potential link between *SLC27A2*^+^ EpCs and other cell types, particularly immune cells, within NP tissue, we conducted cell–cell communication analysis using scRNA-seq data. We found markedly increased interactions between *SLC27A2*^+^ EpC and immune cells, especially T and NK cells, compared with *SLC27A2*^−^ EpC (Fig. 6a). These findings remained consistent when stratified by endotype (Fig. 6b). To pinpoint which immune cell subpopulations interact with *SLC27A2*^+^ EpCs, we further subdivided the T and NK cell clusters and identified a total of ten distinct subtypes (Supplementary Fig. 6a), annotated based on their specific marker gene expression (Supplementary Fig. 6b). We then examined the overall interaction patterns between the ten T cell subtypes and the two EpC types across the different endotypes. In ECRSwNP, we identified 73 significantly strong interaction pathways, while in nECRSwNP, 12 pathways demonstrated relatively strong interactions (Supplementary Fig. 6c). Notably, our analysis highlighted the IL-13 signaling pathway originating from Th2 and ILC2, where secreted IL-13 was predicted to interact with the IL-13 receptor complex (IL-4R + IL-13Rα1; IL-13Rα1) on *SLC27A2*^+^ EpCs in ECRSwNP (Fig. 6c). Interestingly, IL4R and IL13RA1 expression levels were significantly higher in *SLC27A2*^+^ EpCs compared with *SLC27A2*^−^ EpCs (Fig. 6d). However, receptors expression across both endotypes remained relatively consistent, with IL-13 showing pronounced expression in Th2 and ILC2 populations of ECRSwNP (Supplementary Fig. 6d).

IL-13 activity is known to exhibit a robust positive correlation with the prostaglandin E2 (PGE2) activity signature^13,14^. In this context, our analysis identified the prostaglandin signaling pathway originating from EpCs and extending to T and NK cell subpopulations. Prostaglandin signaling was more activated in NP tissues from patients with ECRSwNP compared with those with nECRSwNP (Supplementary Fig. 6c). Notably, *SLC27A2*^+^ EpCs were identified as the predominant regulators driving this pathway relative to *SLC27A2*^−^ EpCs (Supplementary Fig. 6e). These findings suggest a critical role for *SLC27A2*^+^ EpCs in the interplay with Th2 and ILC2 cells via the IL-13 signaling pathway, particularly in the context of ECRSwNP.

Next, we investigated whether IL-4/IL-13 modulate the expression of lipid peroxidation-related genes in EpCs. First, we analyzed a public bulk RNA-seq dataset (GSE268072; 5 resting hNECs and 5 IL-4/IL-13 co-stimulated hNECs) providing transcriptomic profiles of resting hNECs and hNECs co-stimulated with IL-4/IL-13 in an in vitro ALI culture model. IL-4/IL-13 co-stimulation significantly upregulated the expression of lipid peroxidation-related genes, *SLC27A2*, *ALOX15*, *DUOX1* and *NOS2*, while concomitantly downregulating the epithelial junctional protein claudin-encoding genes *CLDN10* and *CLDN9* in hNECs (Fig. 6e). Furthermore, while the expression levels of *IL4R* and *IL13RA1* remained unchanged following IL-4/IL-13 co-stimulation, their expression was sufficient to mediate signaling (Fig. 6f). Notably, the mRNA levels of these receptors were comparable in hNECs from healthy controls and patients with CRSwNP (Fig. 6g). Finally, we sought to explore the changes in gene expression when the IL-4/IL-13 signaling pathway was blocked by dupilumab treatment. We analyzed a public scRNA-seq dataset from a study^10^ investigating the effects of dupilumab treatment. The dataset included samples from two subjects before treatment and three subjects after treatment with dupilumab. Dupilumab is an approved therapy for severe asthma, nasal polyposis and atopic dermatitis that inhibits IL-4 and IL-13 signaling by specifically targeting the shared IL-4Rα subunit of their respective receptor complexes^40–42^. Given the role of IL-13 signaling in regulating lipid peroxidation in EpCs, we hypothesized that inhibiting the IL-13 signaling pathway through dupilumab treatment would downregulate the expression of genes associated with lipid peroxidation in EpCs. Our analysis of the scRNA-seq dataset revealed 12 distinct cell types, identifiable by their respective marker genes (Fig. 6h and Supplementary Fig. 6f). To investigate gene expression changes in the EpC population, we isolated these cells for downstream analysis. In EpCs, dupilumab treatment resulted in the significant downregulation of 241 genes. These downregulated genes were enriched for GO terms related to ATP metabolic processes, oxidative stress responses, cellular responses to lipids and ROS metabolism (Fig. 6i). We validated the decreased expression levels of several lipid peroxidation-related genes, including *SLC27A2*, *ALOX15*, *DUOX1*, *NOS2*, *POR* and *CP* (Fig. 6j). Our findings reveal a critical interplay between IL-4/IL-13-producing Th2/ILC2 cells and *SLC27A2*^+^ EpCs in ECRSwNP. IL-4/IL-13 signaling drives lipid peroxidation and oxidative stress responses in EpCs, which can be mitigated by the IL-4/IL-13 inhibitor dupilumab.

### Association of high *SLC27A2* expression in patients with severe CRSwNP and the effect of *SLC27A2* inhibition on pathogenesis-related markers

**Fig. 7:**
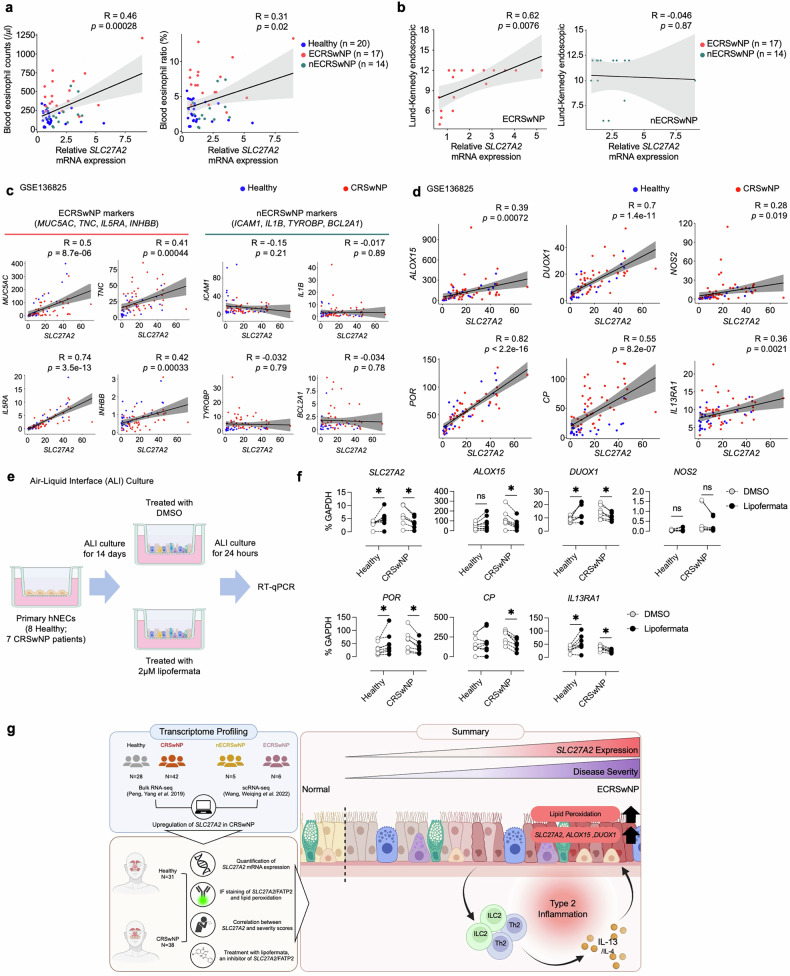
Correlation of elevated *SLC27A2* expression in patients with severe CRSwNP. **a**, A correlation analysis between *SLC27A2* expression and blood eosinophil counts (per µl) and ratio (%) in healthy individuals (*n* = 20) and patients with CRSwNP (*n* = 31). **b**, A correlation analysis between *SLC27A2* expression and clinical severity indicators (Lund–Kennedy endoscopic score) in patients with ECRSwNP (left) and nECRSwNP (right). **c**, The correlation between *SLC27A2* expression and markers of eosinophilic (*MUC5AC*, *TNC*, *IL5RA* and *INHBB*) and non-eosinophilic (*ICAM1*, *IL1B*, *TYROBP* and *BCL2A1*) CRSwNP in the GSE136825 dataset. **d**, The correlation between *SLC27A2* expression with *ALOX15*, *DUOX1*, *NOS2*, *POR*, *CP* and *IL13RA1* expression. **e**, Primary hNECs were cultured using an ALI method and subsequently treated with lipofermata for 24 h. RNA extraction was performed on these cells, followed by RT–qPCR analysis. **f**, mRNA expression of candidate genes in ALI-cultured hNECs derived from healthy individuals (*n* = 8) and patients with CRSwNP (*n* = 7). Cells were treated with or without 2 µM lipofermata for 24 h, and mRNA levels were assessed via RT–qPCR, normalized to GAPDH mRNA levels. **g**, A schematic representation providing an overview of key study findings. Spearman correlation coefficients (*R*) and associated *P* values are depicted. *P* values were derived from multiple Wilcoxon matched-pairs signed-rank tests. *P < 0.05, **P < 0.01, ***P < 0.001 and ****P < 0.0001.

We examined whether the expression of *SLC27A2* clinically correlates with type 2 inflammation, as the crosstalk between *SLC27A2*^+^ EpCs and Th2/ILC2 is closely linked to the IL-13 signaling pathway, a key driver of type 2 inflammation. As anticipated, eosinophil counts, a hallmark of type 2 inflammation, displayed a robust correlation with *SLC27A2* mRNA expression (Fig. 7a). Notably, *SLC27A2* mRNA expression demonstrated a significant positive correlation with Lund–Kennedy endoscopic scores in patients with ECRSwNP, whereas no such correlation was observed in patients with nECRSwNP. Specifically, higher *SLC27A2* mRNA expression was associated with more severe endoscopic findings in ECRSwNP (Fig. 7b). To further interrogate the molecular associations of SLC27A2, we analyzed bulk RNA-seq datasets. While ECRSwNP markers (*MUCA5AC*, *TNC*, *IL5RA* and *INHBB*)^11,43–46^ demonstrated significant correlations, no such relationship was observed with nECRSwNP markers (*ICAM1*, *IL1B*, *TYROBP* and *BCL2A1*)^47^ associated with neutrophil infiltration (Fig. 7c and Supplementary Fig. 7a). Among lipid peroxidation-related genes, *ALOX15*, *DUOX1*, *NOS2* and *POR* exhibited a positive correlation with *SLC27A2*. However, while a significant correlation was observed for *CP* in the GSE136825 dataset, only a trend was evident in the GSE179269 dataset. Additionally, a positive correlation between *IL13RA1*, a receptor for IL-13, and *SLC27A2* was confirmed in both datasets (Fig. 7d and Supplementary Fig. 7b). No correlation was observed between genes associated with lipid peroxidation (*ACSL4*, *LPCAT3* and *LCN2*) and genes related to the IL-4/IL-13 signaling pathway (*IL4*, *IL13* and *IL4R*) (Supplementary Fig. 7c). Our comprehensive analysis reveals that SLC27A2 exhibits significant molecular associations with type 2 inflammation, characterized by robust correlations with blood eosinophil counts, disease severity, ECRSwNP markers, lipid peroxidation-related genes and IL-13 receptor expression.

To explore the clinical significance of our findings, we evaluated the effect of *SLC27A2*/FATP2 inhibition on disease severity using an in vitro ALI culture model of hNECs. ALI cultures were established using hNECs derived from eight healthy individuals and seven patients with CRSwNP. To determine the impact of *SLC27A2*/FATP2 inhibition, we treated the ALI cultures with 2 μM lipofermata, a specific inhibitor of *SLC27A2*/FATP2, and evaluated the expression levels of *SLC27A2* and pathogenesis-related markers that exhibited a positive correlation with *SLC27A2* (Fig. 7e). In NP-derived EpCs, lipofermata treatment led to a significant decrease in mRNA expression levels of *SLC27A2*, *ALOX15*, *DUOX1*, *POR*, *CP* and *IL13RA1*. However, no change was observed in the expression of *NOS2* (Fig. 7f). Interestingly, in EpCs derived from healthy individuals, no significant changes in the expression of these genes were detected upon lipofermata treatment (Fig. 7f). These findings suggest that the inhibition of *SLC27A2* specifically attenuates the expression of pathogenesis-related markers in hNECs from patients with CRSwNP.

In summary, integrated transcriptomic analysis and experimental validation identified significant upregulation of lipid peroxidation and *SLC27A2*/FATP2 in the NP epithelium of patients with CRSwNP. Notably, *SLC27A2*^+^ EpCs were shown to respond to IL-13 signaling derived from Th2/ILC2. Furthermore, *SLC27A2* expression demonstrated a strong positive correlation with type 2 inflammatory signatures and disease severity in patients with ECRSwNP (Fig. 7g), highlighting its potential as both a marker of disease severity and a promising therapeutic target.

## Discussion

CRS is a complex and multifactorial inflammatory condition of the sinonasal mucosa, often accompanied by the presence of NPs. One key aspect of the pathogenesis of this disease is the dysfunction of EpCs that line the sinonasal cavity. Numerous studies have highlighted the importance of EpC dysfunction in CRSwNP, as persistent inflammatory insults disrupt the epithelial cellular ecosystem, subsequently provoking exacerbated inflammatory responses^10,11,48^. In recent years, multiple studies have identified dysregulated metabolic processes within the NP epithelium and highlighted their pathogenic relevance^13–15,20,24,49,50^. These findings suggest that metabolic alterations in the EpCs may contribute to the development and progression of CRSwNP. However, the precise mechanisms by which changes in metabolic gene expression led to the dysregulation of EpCs in CRSwNP are still not fully understood.

Our study aimed to investigate the role of altered metabolic processes, particularly those related to lipid metabolism, in the pathogenesis of CRSwNP. By employing a comprehensive transcriptomic approach, we sought to identify key metabolic genes and pathways that contribute to EpC dysfunction in CRSwNP and to elucidate the potential mechanisms underlying their pathogenic effects. Our analysis revealed aberrant expression of genes related to lipid metabolic pathways in EpCs and myeloid cells within the NP tissue, suggesting a potential link between disrupted lipid homeostasis and CRSwNP pathogenesis. This finding aligns with the higher incidence of CRS in individuals with metabolic syndrome, supporting the hypothesis that metabolic dysregulation plays a role in the development of nasal polyposis^51,52^. In addition, increased levels of unsaturated fatty acids and uric acid have been observed in different CRSwNP subtypes, suggesting a metabolic milieu that promotes neutrophilic and eosinophilic inflammation^52–54^. These findings indicate that alterations in lipid metabolism may contribute to shaping the inflammatory microenvironment within the NP tissue, potentially driving the development and progression of CRSwNP.

Our results revealed an elevated lipid peroxidation signal, predominantly localized to the epithelial regions, in the NP tissues of patients with CRSwNP compared with normal control tissues. This observation is consistent with previous studies that have demonstrated markedly elevated levels of 4-hydroxynonenal and 3-nitrotyrosine, indicators of oxidative stress, in NP tissues compared with control nasal tissues^24^. Moreover, a distinct upregulation of genes intimately associated with ferroptosis, a form of regulated cell death driven by lipid peroxidation, has been reported in NPs^49^. Our study identified *ALOX15* and *SLC27A2* as potential candidates for inducing lipid peroxidation in the NP epithelium, with both genes exhibiting significantly elevated expression in patients with CRSwNP. Notably, *ALOX15* is already recognized as a prominent biomarker with a marked increase in ECRSwNP^10,11,19,21,55^. The co-localization of *SLC27A2*/FATP2 with lipid peroxidation markers showed a positive correlation in stained area percentages, further supporting its potential role in lipid peroxidation within the NP epithelium. Interestingly, we observed comparable levels of lipid peroxidation and *SLC27A2*/FATP2 expression in both ECRSwNP and nECRSwNP. This finding suggests that *SLC27A2* may contribute to lipid peroxidation through an endotype-independent mechanism.

FATP2 functions as a crucial facilitator for the uptake and integration of polyunsaturated fatty acids (PUFAs) into phospholipids, particularly arachidonic acid (AA) and adrenic acid (AdA)^36^. The absence of FATP2 in cells results in reduced levels of arachidonoyl-phosphatidylethanolamine^51^, highlighting the critical role of FATP2 in synthesizing AA-coenzyme A (CoA) and AdA-CoA, both essential precursors in the lipid peroxidation pathway^38,39,56,57^. Positioned at the nexus of PUFA uptake and conversion, FATP2 operates at the apical membrane and endoplasmic reticulum, facilitating the transformation of AA and AdA into their CoA derivatives^57^. This process is complemented by ACSL4 and LPCAT3 within the endoplasmic reticulum, promoting the activation and incorporation of AA/AdA-CoA into phosphatidylethanolamine. The subsequent enzymatic action of 15-lipoxygenase, encoded by *ALOX15*, along with the contribution of POR, leads to an accumulation of lipid hydroperoxides, which precipitates an increase in lipid peroxidation and potential cellular injury^57,58^. Our investigation revealed elevated expression of *ACSL4*, *LPCAT3*, *ALOX15* and *POR* in *SLC27A2*^+^ EpC, compared with *SLC27A2*^−^ EpC from NP tissue. Furthermore, the upregulation of *DUOX1*, *NOS2*, *CP* and *LCN2*, genes implicated in lipid peroxidation, corroborated these findings. Our findings suggest FATP2 can contribute to the accumulation of lipid hydroperoxides and the subsequent increase in lipid peroxidation. This, in turn, may lead to cellular damage and dysfunction, potentially contributing to the impairment of epithelial barrier function and the perpetuation of inflammatory processes in CRSwNP.

In ECRSwNP, *SLC27A2*^*+*^ EpCs exhibits a relatively stronger response to IL-13 signals from Th2 and ILC2 populations compared with *SLC27A2*^−^ EpCs. Previous studies have shown that exposure of cultured differentiated hNECs to IL-13 leads to the loss of ciliated cells and the induction of ETS transcription factor Spdef via STAT6 activation, consequently suppressing FOXA2 and upregulating *ALOX15*, a lipid peroxidation-induced gene^59,60^. Interestingly, inhibition of IL-13 signaling via dupilumab, a monoclonal antibody targeting the IL-4 receptor α subunit, leads to the downregulation of genes associated with oxidative stress and ROS metabolism. Notably, the expression of *SLC27A2*, *ALOX15*, *DUOX1*, *NOS2*, *POR* and *CP* genes implicated in lipid peroxidation, is also diminished upon blocking IL-13. These findings suggest that IL-13 signaling plays a crucial role in the regulation of lipid peroxidation and oxidative stress in the NP epithelium of patients with ECRSwNP.

The pronounced expression of *SLC27A2* in ciliated cells raises the intriguing possibility that the observed loss of these cells in ECRSwNP may be attributable to *SLC27A2*/FATP2-mediated lipid peroxidation. Given the critical role of ciliated cells in maintaining mucociliary clearance and epithelial barrier function, the loss of these cells due to FATP2-mediated lipid peroxidation could contribute significantly to the pathogenesis of ECRSwNP. However, further investigation is warranted to elucidate the direct contributions of this relationship and to determine the precise mechanisms by which *SLC27A2*/FATP2 and IL-13 signaling interact to promote ciliated cell loss and epithelial dysfunction in ECRSwNP.

Previous studies have demonstrated that IL-13 not only drives nasal epithelial remodeling, but also exhibits a strong association with the PGE2 activation signature^13,14^. IL-13-programmed airway tuft cells have been identified as a significant source of PGE2, with a positive correlation observed between their respective transcriptional profiles. Conversely, IL-13 stimulation has been shown to negatively correlate with the transcriptional signatures of ciliated cells. These findings suggest that IL-13 may simultaneously induce PGE2 production through tuft cell activation while compromising the ciliated cell population, thereby perpetuating the inflammatory milieu and epithelial dysregulation in nasal polyposis^13,14^. In addition to PGD2, another prostanoid, has been implicated in the recruitment and activation of Th2, ILC2 and eosinophils^61^. Our cell–cell interaction analysis using scRNA-seq data confirmed that *SLC27A2*^+^ EpCs, which are potent recipients of IL-13 signals, propagate prostaglandin signals more robustly to various subtypes of T cells, ILCs and NK cells compared with *SLC27A2*^−^ EpCs. This suggests that enhanced prostaglandin signaling from *SLC27A2*^+^ EpCs to immune cells in the NP microenvironment may have significant implications for the pathogenesis of CRSwNP.

These findings suggest a compelling mechanistic link between IL-13, a key mediator of type 2 inflammation, and *SLC27A2*/FATP2 expression. This hypothesis is further supported by clinical observations, as *SLC27A2* mRNA expression levels correlate positively with blood eosinophil counts, eosinophil ratio and disease severity in patients with ECRSwNP. However, no significant association was observed when evaluating patients with nECRSwNP, highlighting potential differences in disease mechanisms between these subtypes. Consistently, *SLC27A2* expression demonstrated robust correlations with key ECRSwNP molecular markers, including *ALOX15*, *IL13RA1*, *MUC5AC*, *TNC*, *IL5RA* and *INHBB*. Conversely, the marker showed no significant associations with neutrophilic or nECRSwNP markers, such as *ICAM1*, *IL1B*, *TYROBP* and *BCL2A1*, further delineating the molecular specificity of *SLC27A2* in eosinophilic inflammatory pathways. These findings suggest that lipid peroxidation in ECRSwNP may be driven predominantly by *SLC27A2*/FATP2 activity. However, despite elevated FATP2 expression in nECRSwNP, the relatively low expression of *ALOX15* implies that lipid peroxidation in nECRSwNP may be regulated through pathways independent of lipid mediators. This indicates the need for future studies to explore alternative pathways, potentially driven by iron or thiol metabolism, to identify molecular markers governing lipid peroxidation and ferroptosis in nECRSwNP.

Mechanistically, Lipofermata functions as a noncompetitive inhibitor, specifically suppressing the PUFA transport activity of *SLC27A2*/FATP2, thereby reducing its functionality and expression levels^62–64^. These effects are more pronounced in cells with high *SLC27A2* expression.^62,63^ Lipofermata also impacts the PI3K/AKT/mTOR signaling pathway, which regulates essential cellular processes such as proliferation, survival and metabolism.^64^ By inhibiting FATP2 activity and downregulating its mRNA expression, lipofermata blocks fatty acid uptake, leading to alterations in intracellular lipid metabolism. Consequently, the observed downregulation of *ALOX15*, *DUOX1*, *POR*, *CP* and *IL13RA1* mRNA levels in cultured hNECs treated with lipofermata is probably attributed to indirect effects driven by changes in lipid metabolism mechanisms rather than direct molecular interactions. Collectively, the reduction in *SLC27A2*/FATP2 expression induced by lipofermata appears to modulate gene expression indirectly through shifts in lipid metabolism and the PI3K/AKT/mTOR pathway. These findings highlight the potential of targeting *SLC27A2* as a therapeutic strategy for ECRSwNP by attenuating lipid peroxidation and IL-13 signaling in the NP epithelium.

In conclusion, our study provides novel insights into the role of *SLC27A2* in the pathogenesis of ECRSwNP, highlighting its potential as a biomarker and therapeutic target. *SLC27A2* may contribute to lipid peroxidation in the NP epithelium, which exhibits dysregulated lipid metabolic processes. *SLC27A2*^+^ EpCs, potent recipients of IL-13 signals from Th2/ILC2, actively propagate prostaglandin signaling and promote aberrant mucociliary dysfunction and inflammation. Furthermore, *SLC27A2* expression positively correlates with disease severity in patients with ECRSwNP, and its inhibition suppresses the expression of identified pathogenesis-related genes. Our findings suggest that targeting *SLC27A2* and its associated pathways, such as lipid peroxidation and IL-13 signaling, may offer new opportunities for more nuanced patient stratification and tailored therapeutic approaches in severe cases of CRSwNP. Further clinical and mechanistic studies are necessary to validate these findings and explore the potential of *SLC27A2*/FATP2-targeted therapies in the management of ECRSwNP.

## Supplementary information

Supplementary information

Supplementary Table 2.

Supplementary Table 3.

Supplementary Table 4.
