# Supplementary material for: SLC27A2 marks lipid peroxidation in nasal epithelial cells driven by type 2 inflammation in chronic rhinosinusitis with nasal polyps
Source: Exp Mol Med. 2025 Apr 7;57(4):856–71. doi: 10.1038/s12276-025-01440-1 (PMC12045986; doi:10.1038/s12276-025-01440-1)
Supplement: Supplementary file 1 — Supplementary information [file 12276_2025_1440_MOESM1_ESM.pdf]

SLC27A2 marks lipid peroxidation in nasal epithelial cells driven by type 2 inflammation in chronic rhinosinusitis with nasal polyps

Jaewoo Park[1], Jung Yeon Jang[2], Jeong Heon Kim[2], Se Eun Yi[2], Yeong Ju Lee[2], Myeong Sang Yu[2], Yoo-Sam Chung[2], Yong Ju Jang[2], Ji Heui Kim[2,3] and Kyuho Kang[1,3]

[1] Department of Biological Sciences and Biotechnology, Chungbuk National University, Cheongju 28644, Republic of Korea

[2] Department of Otorhinolaryngology – Head and Neck Surgery, Asan Medical Center, University of Ulsan College of Medicine, Seoul, Republic of Korea

[3] Correspondence: Ji Heui Kim, jhkim0217@amc.seoul.kr; Kyuho Kang, kangk@cbnu.ac.kr

Supplementary data

Supplementary Table 1. Primers for mRNA expression

**Target gene**		**Sequence (5’-3’)**
GAPDH	Forward	GACCCCTTCATTGACCTC
	Reverse	GCTAAGCAGTTGGTGGTG
SLC27A2	Forward	TACTCTTGCCTTGCGGACTAA
	Reverse	CCGAAGCAGTTCACCGATATAC
ALOX15	Forward	TGGAAGGACGGGTTAATTCTGA
	Reverse	GCGAAACCTCAAAGTCAACTCT
DUOX1	Forward	CCTGGCTCTAGCATGGACAC
	Reverse	CTGCACCTCCCACGAAATG
NOS2	Forward	GCTCTACACCTCCAATGTGACC
	Reverse	CTGCCGAGATTTGAGCCTCATG
POR	Forward	GGGATGCGAGGCATGTCAG
	Reverse	CAGGGCGTTGTCGATCTCT
CP	Forward	GGGCCATCTACCCTGATAACA
	Reverse	TTAAAGGTCCGATGAGTCCTGA
IL13RA1	Forward	GTCCCAGTGTAGCACCAATGA
	Reverse	GCTCAGGTTGTGCCAAATGC

Supplementary Fig. 1

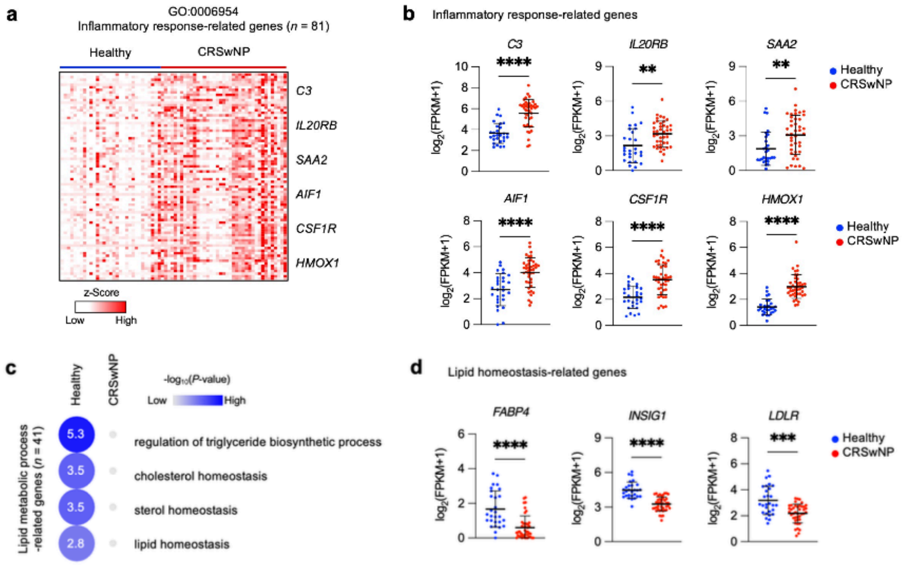

Supplementary Fig. 2

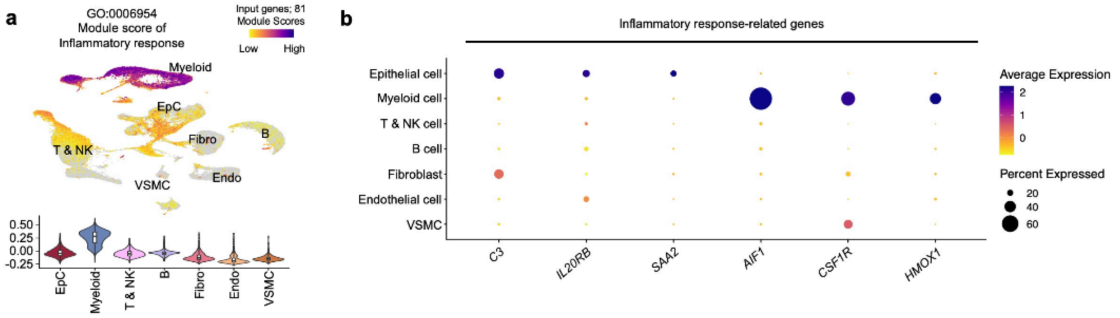

Supplementary Fig. 3

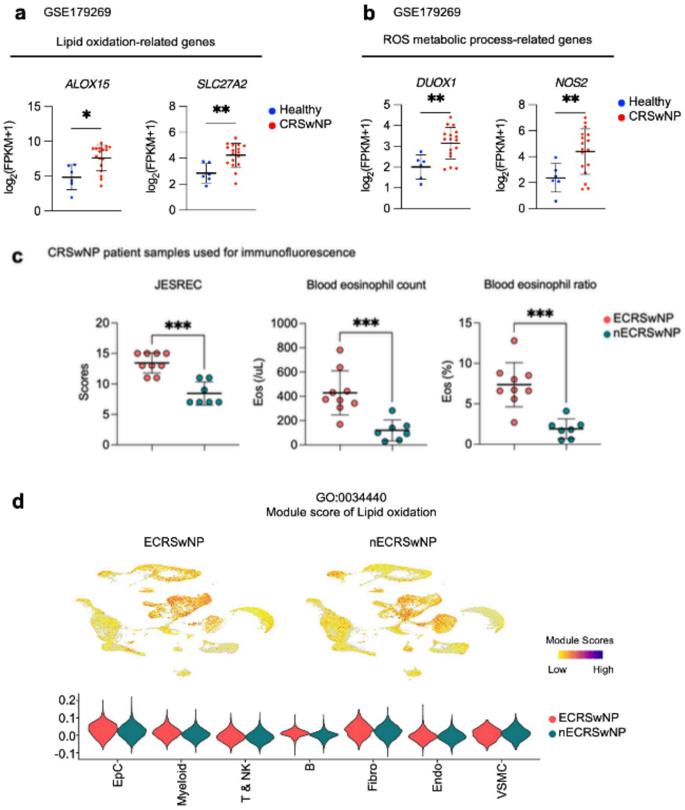

Supplementary Fig. 4

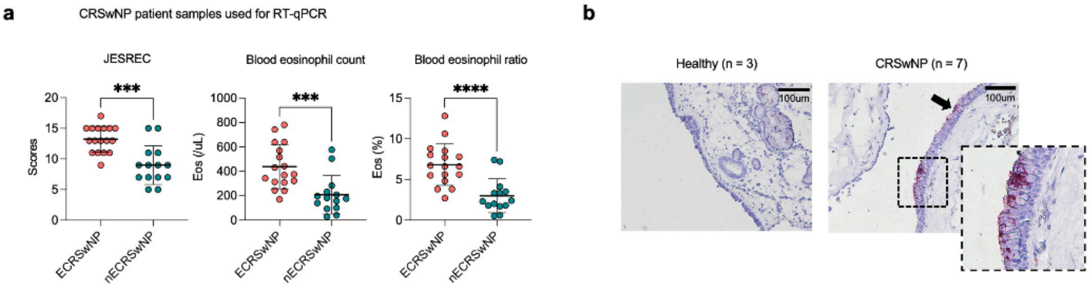

Supplementary Fig. 5

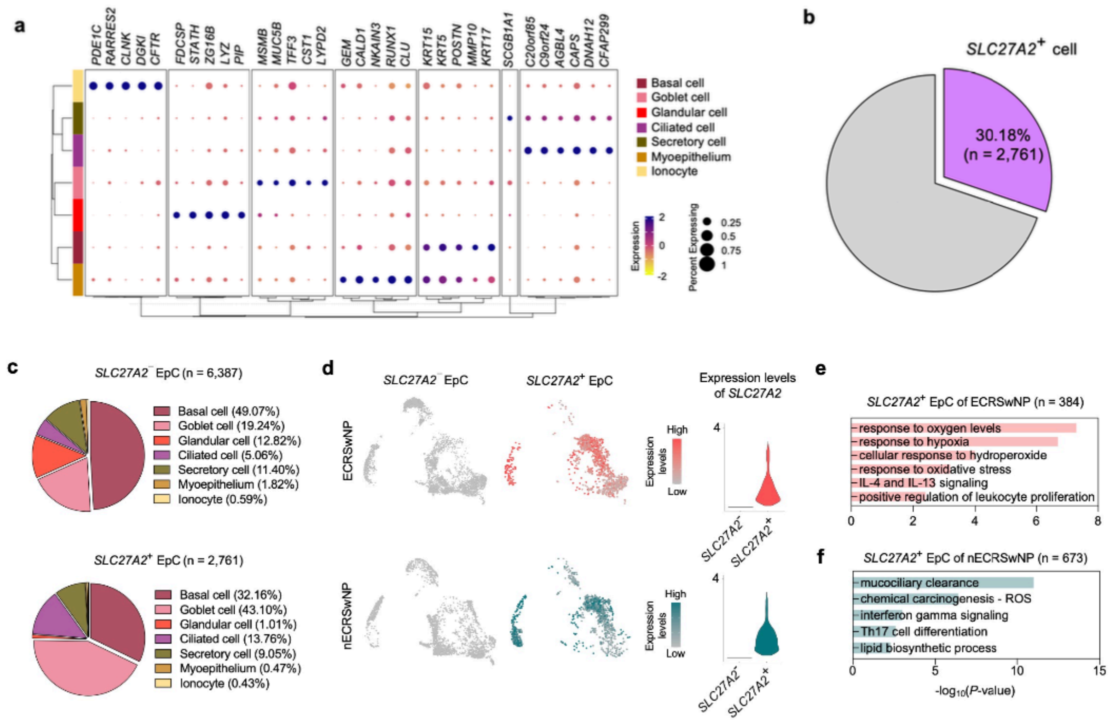

Supplementary Fig. 6

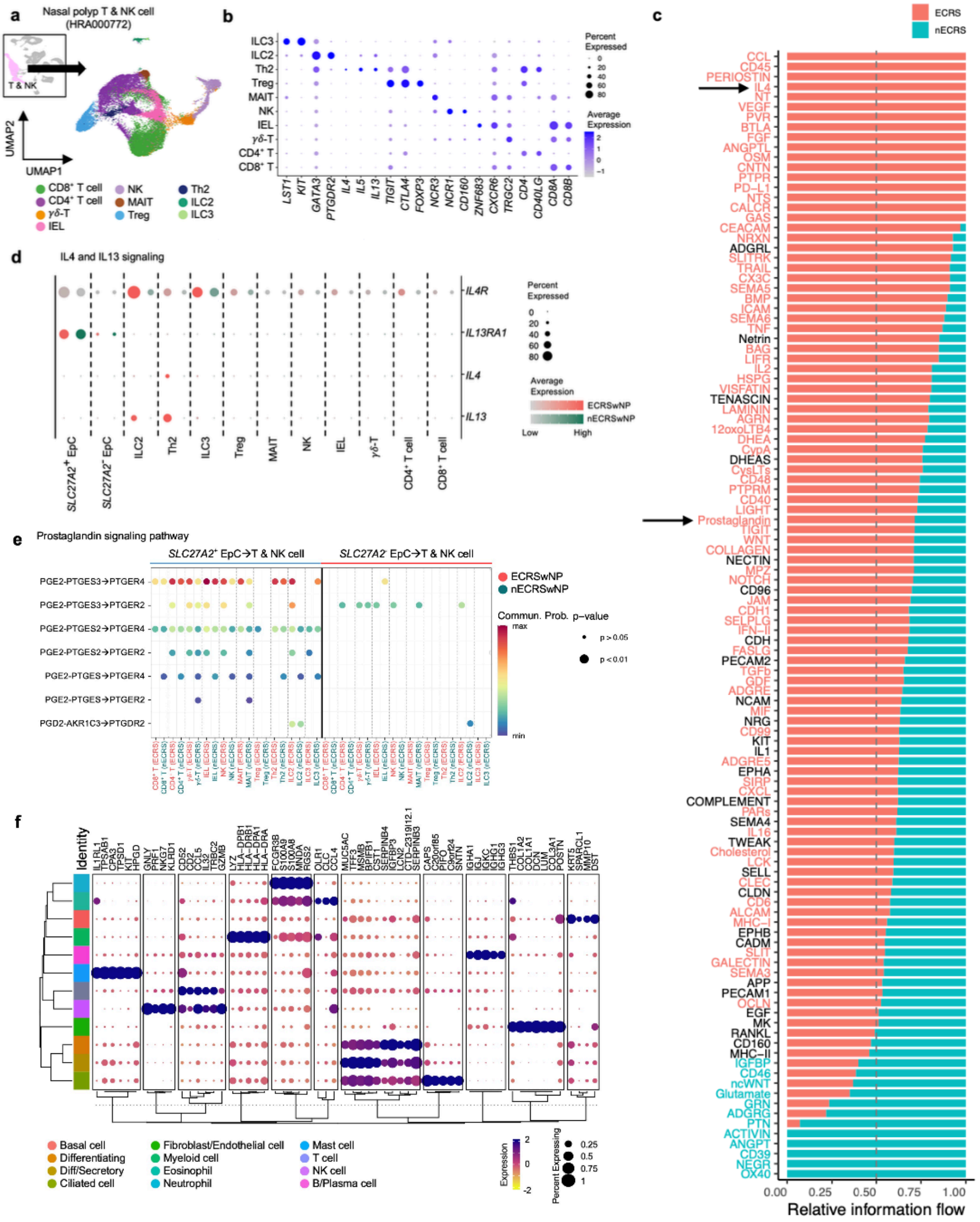

Supplementary Fig. 7

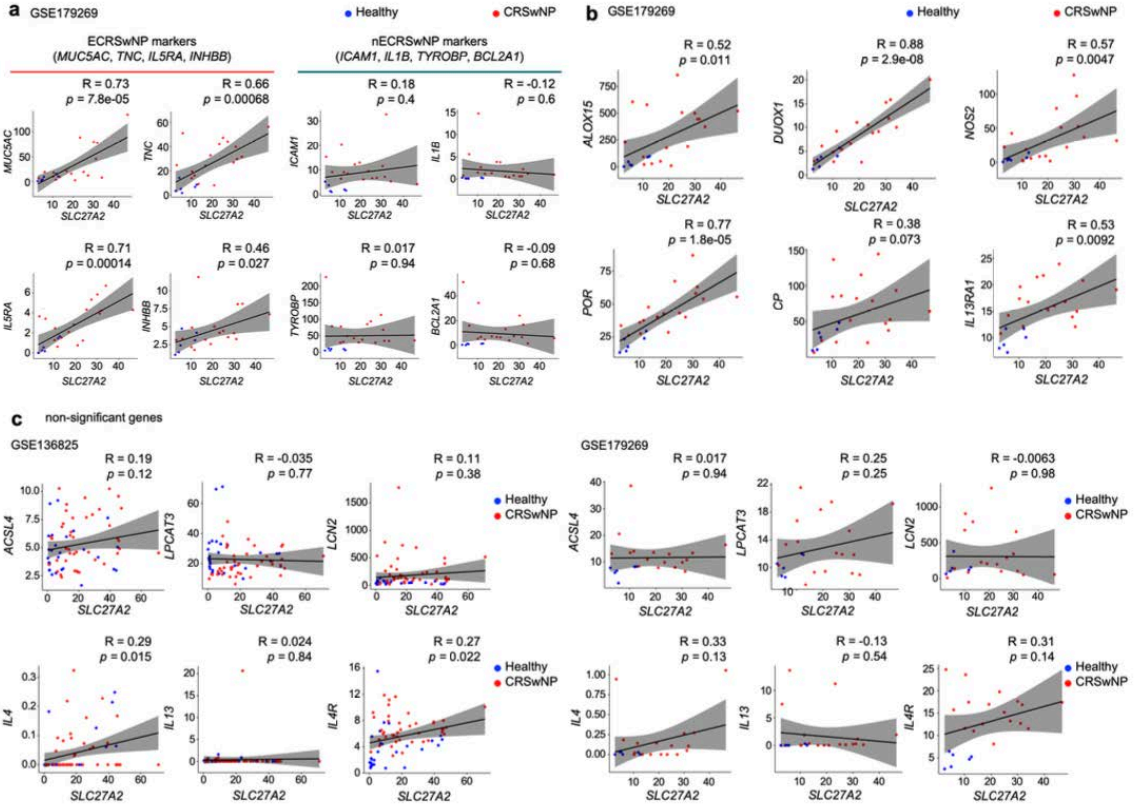

Supplementary Figure Legends

Supplementary Fig. 1 Transcriptomic expression differences between healthy controls and CRSwNP patients.

a Heatmaps showing upregulated genes in CRSwNP involved in inflammatory response. Values are z-Scores. **b** Expression levels of representative genes associated with inflammatory response in healthy controls and CRSwNP patients. **c** GO analysis of the 41 downregulated genes associated with lipid metabolic processes from the 419 upregulated genes in CRSwNP. **d** Expression levels of representative genes associated with lipid homeostasis in healthy controls and CRSwNP patients. Error bars represent mean values ± SD. *P*-values determined by Welch's t-test. * $P < 0.05$, ** $P < 0.01$, *** $P < 0.001$, **** $P < 0.0001$.

Supplementary Fig. 2 Cell type-specific expression patterns of genes involved in inflammatory responses in nasal polyp tissues.

a UMAP (left) and violin plots (right) showing the single-cell module scores for inflammatory response across different cell types in CRSwNP nasal polyp tissues. **b** Dot plots depicting the expression patterns of representative genes associated with inflammatory response in different cell types from CRSwNP nasal polyp tissues. Each dot represents the percentage of cells expressing the given gene within each cell type, and the color scale indicates the average expression level.

Supplementary Fig. 3 *SLC27A2* expression coincides with lipid peroxidation in nasal polyp epithelium.

a, b Comparison of expression levels of representative genes involved in lipid peroxidation and ROS metabolic process identified using the GSE179269 dataset. **c** Comparative analysis of JESREC Scores, Blood Eosinophil Count (cells/μl), and Tissue Eosinophil Ratio (%) in

discriminating between ECRSwNP (n = 9) and nECRSwNP (n = 7) among CRSwNP samples. **d** UMAP (top) and violin plots (bottom) showing the single-cell module scores for lipid oxidation across different cell types in nasal polyp tissues from ECRSwNP and nECRSwNP. Error bars represent mean values ± SD. *P*-values determined by Welch's t-test. * $P < 0.05$, ** $P < 0.01$, *** $P < 0.001$, **** $P < 0.0001$.

Supplementary Fig. 4 Increased expression of *SLC27A2*/FATP2 in nasal polyp epithelium. **a** Comparative analysis of JESREC Scores, Blood Eosinophil Count (cells/μl), and Tissue Eosinophil Ratio (%) in discriminating between ECRSwNP (n = 17) and ECRSwNP (n = 14) among CRSwNP samples. **b** ORO staining of Healthy (n = 3) and CRSwNP (n = 7). Error bars represent mean values ± SD. *P*-values determined by Welch's t-test. * $P < 0.05$, ** $P < 0.01$, *** $P < 0.001$, **** $P < 0.0001$. Scale bars: 50μm and 100μm.

Supplementary Fig. 5 Transcriptomic heterogeneity of *SLC27A2*$^{+}$ NP EpCs across endotypes.

a Dot plot of gene expression displaying major markers for the 7 subtypes of epithelial cell. Each dot represents the percentage of cells expressing the given gene within each cell type, and the color scale indicates the average expression level. **b** Pie graph shows the percentage of *SLC27A2*$^{+}$ EpC. **c** Pie graph shows the percentages of each 7 subtype of epithelial cell in total *SLC27A2*$^{-}$ EpC (upper pie graph) and *SLC27A2*$^{+}$ EpC (lower pie graph) from CRSwNP patients. **d** UMAP plots depicting *SLC27A2*$^{-}$ and *SLC27A2*$^{+}$ EpC in nasal polyps from 6 ECRSwNP (top) and 5 nECRSwNP (bottom). *SLC27A2* expression is color-indicated (left). Violin plot showing the expression levels of *SLC27A2*$^{-}$ and *SLC27A2*$^{+}$ EpC (right). **e** GO analysis of 384 genes expressed more abundantly in *SLC27A2*$^{+}$ EpC than *SLC27A2*$^{-}$EpC in ECRSwNP. **f**, GO analysis of 673 genes expressed more abundantly in *SLC27A2*$^{+}$ EpC than

SLC27A2⁻ EpC in nECRSwNP.

Supplementary Fig. 6 Distinct interactions between *SLC27A2*$^+$ NP EpCs and Th2/ILC2.

a UMAP displaying 23,715 T & NK cells from 11 CRSwNP categorized into 10 subsets (CD8+ T cell; CD4+ T cell; $\gamma\delta$-T cell; IEL, Intraepithelial lymphocytes; NK cell, Natural killer cell; MAIT, Mucosal-associated invariant T cell; Treg, Regulatory T cell; Th2, T helper 2 cell; ILC2, Group 2 Innate Lymphoid Cell; ILC3, Group 3 Innate Lymphoid Cell). **b** Dot plot of representative gene expression levels mapped onto each T & NK cell subsets. **c** Bar chart showing important signaling pathways between NP EpC and T & NK cell subtypes based on the differences in overall information flow within the inferred network between ECRSwNP and nECRSwNP. **d** The dot plot depicts the relative expression of key ligands (IL4, IL13) and their receptors (IL4R, IL13RA1) in different cell populations, including EpC and T & NK cell subtypes. **e** Comparison of the significant ligand-receptor pairs between ECRSwNP and nECRSwNP, which contribute to the prostaglandin signaling from EpC to T & NK cell subpopulations. Dot color reflects communication probabilities and dot size represents computed p-values. Empty space means the communication probability is zero. **f** Dot plot of gene expression displaying major markers for the 12 subtypes of public scRNA-seq dataset (dupilumab data from Ordovas-Montanes et al., 2018). Each dot represents the percentage of cells expressing the given gene within each cell type, and the color scale indicates the average expression level.

Supplementary Fig. 7 Correlation analysis of *SLC27A2* with candidate genes.

a Correlation between *SLC27A2* expression and markers of eosinophilic (*MUC5AC*, *TNC*, *IL5RA*, and *INHBB*) and non-eosinophilic (*ICAM1*, *IL1B*, *TYROBP*, and *BCL2A1*) CRSwNP in the GSE179269 dataset. **b** Association of *SLC27A2* expression with *ALOX15*, *DUOX1*,

NOS2, *POR*, *CP*, and *IL13RA1* expression in GSE179269 dataset. **c** Association of *SLC27A2* expression with *ACSL4*, *LPCAT3*, *LCN2*, *IL4*, *IL13*, and *IL4R* expression in GSE136825 (left) and GSE179269 dataset (right). Spearman correlation coefficients (R) and associated *P*-value.
